# Severe maternal morbidity surveillance: Monitoring pregnant women at high risk for prolonged hospitalisation and death

**DOI:** 10.1111/ppe.12574

**Published:** 2019-08-12

**Authors:** Susie Dzakpasu, Paromita Deb‐Rinker, Laura Arbour, Elizabeth K. Darling, Michael S. Kramer, Shiliang Liu, Wei Luo, Phil A. Murphy, Chantal Nelson, Joel G. Ray, Heather Scott, Michiel VandenHof, K. S. Joseph

**Affiliations:** ^1^ Maternal, Child and Youth Health Division, Centre for Surveillance and Applied Research Public Health Agency of Canada Ottawa ON Canada; ^2^ Department of Medical Genetics University of British Columbia Victoria BC Canada; ^3^ Department of Obstetrics and Gynecology McMaster University Hamilton ON Canada; ^4^ Department of Pediatrics and of Epidemiology and Biostatistics McGill University Montreal QC Canada; ^5^ Perinatal Program of Newfoundland and Labrador St. John’s NFL Canada; ^6^ Department of Medicine University of Toronto Toronto ON Canada; ^7^ Department of Obstetrics and Gynaecology Dalhousie University Halifax NS Canada; ^8^ Department of Obstetrics and Gynaecology University of British Columbia Vancouver BC Canada

**Keywords:** maternal mortality, severe maternal morbidity, surveillance

## Abstract

**Background:**

There is no international consensus on the definition and components of severe maternal morbidity (SMM).

**Objectives:**

To propose a comprehensive definition of SMM, to create an empirically justified list of SMM types and subtypes, and to use this to examine SMM in Canada.

**Methods:**

Severe maternal morbidity was defined as a set of heterogeneous maternal conditions known to be associated with severe illness and with prolonged hospitalisation or high case fatality. Candidate SMM types/subtypes were evaluated using information on all hospital deliveries in Canada (excluding Quebec), 2006‐2015. SMM rates for 2012‐2016 were quantified as a composite and as SMM types/subtypes. Rate ratios and population attributable fractions (PAF) associated with overall and specific SMM types/subtypes were estimated in relation to length of hospital stay (LOS > 7 days) and case fatality.

**Results:**

There were 22 799 cases of SMM subtypes (among 1 418 545 deliveries) that were associated with a prolonged LOS or high case fatality. Between 2012 and 2016, the composite SMM rate was 16.1 (95% confidence interval [CI] 15.9, 16.3) per 1000 deliveries. Severe pre‐eclampsia and HELLP syndrome (514.6 per 100 000 deliveries), and severe postpartum haemorrhage (433.2 per 100 000 deliveries) were the most common SMM types, while case fatality rates among SMM subtypes were highest among women who had cardiac arrest and resuscitation (241.1 per 1000), hepatic failure (147.1 per 1000), dialysis (67.6 per 1000), and cerebrovascular accident/stroke (51.0 per 1000). The PAF for prolonged hospital stay related to SMM was 17.8% (95% CI 17.3, 18.3), while the PAF for maternal death associated with SMM was 88.0% (95% CI 74.6, 94.4).

**Conclusions:**

The proposed definition of SMM and associated list of SMM subtypes could be used for standardised SMM surveillance, with rate ratios and PAFs associated with specific SMM types/subtypes serving to inform clinical practice and public health policy.

**Synopsis**

**Study question**

- To define severe maternal morbidity (SMM), to create an empirically justified list of SMM types and subtypes, and to use this list to examine SMM rates in Canada.

**What is already known**

- Previous lists of SMM components exclude some important SMM types and subtypes and include some SMM types and subtypes that do not reflect severe morbidity.

**What this study adds**

- SMM is defined as a set of heterogeneous maternal conditions known to be associated with severe illness and prolonged hospitalisation or high case fatality
- A set of severely morbid maternal conditions was identified based on a priori clinical knowledge, prolonged length of hospital stay, high case fatality, and expert consensus, and this should help improve surveillance and benchmarking of SMM in Canada and other high‐income countries.

## 1 INTRODUCTION

Substantial changes in maternity care were introduced in industrialised countries in the middle decades of the 20th century, including improvements in the organisation of obstetric services and widespread use of antibacterial agents, ergometrine, and blood transfusion.^1^, ^2^, ^3^ The substantial reduction in maternal mortality that followed led to a perception that pregnancy and childbirth had become safe and mostly risk‐free. However, maternal mortality remains a concern among vulnerable subpopulations even in high‐income countries, and severe maternal morbidity (SMM) is recognised to affect an important fraction of women.^3^, ^4^, ^5^, ^6^, ^7^

Although maternal death represents a more extreme and serious outcome than severe maternal illness, its rarity resulted in SMM becoming an important focus for public health surveillance and epidemiologic investigation in high‐income countries.^3^, ^6^, ^7^, ^8^ Recent changes in maternal characteristics in such countries, including increases in age and pre‐pregnancy weight, have raised new concerns about temporal trends in SMM.^9^, ^10^ Reviews of SMM cases show that, as with maternal death, the most common preventable factor is suboptimal care, including failures in diagnosis and delays in treatment.^11^, ^12^, ^13^, ^14^ The World Health Organization has recommended that maternal health surveillance focus not only on maternal mortality but also on severe acute maternal morbidity, in order to identify priorities for intervention.^15^

The World Health Organization defines *severe maternal complications* as ‘potentially life‐threatening conditions’, *maternal near‐miss* as ‘a woman who nearly died but survived a complication that occurred during pregnancy, childbirth or within 42 days of termination of pregnancy’, and *severe maternal outcomes* as maternal near‐miss cases and maternal deaths (per WHO terminology, *severe acute maternal morbidity* is synonymous with maternal near‐miss).^15^, ^16^ The WHO definition of near‐miss (or severe acute maternal morbidity) notwithstanding, there is little international consensus on the components of SMM, and studies on SMM typically include variable lists of maternal diseases, interventions, and organ failure types (without clear specification of how these meet a prespecified definition).^4^, ^5^, ^6^, ^7^ Large differences in SMM conditions included in different studies lead to incomparable SMM frequencies and an inability to benchmark population rates of SMM. The objectives of this study were to propose a comprehensive definition of SMM, to use the definition to create an empirically justified list of component SMM types and subtypes, and to use this list to examine SMM rates in Canada. The proposed definition of SMM, and its components, could serve to spur efforts towards creating an international consensus for SMM surveillance and for benchmarking maternal health outcomes in populations.

## 2 METHODS

### 2.1 Historical note

The Canadian Perinatal Surveillance System (CPSS) began monitoring SMM approximately 15 years ago,^17^, ^18^ using diagnosis and intervention codes (based on the International Classification of Diseases and Related Health Problems, 9th Revision (ICD‐9) and the Canadian Classification of Procedures) in hospitalisation data. The list of conditions that constituted SMM was revised in 2010 after a formal assessment of the diagnoses and procedures available in the Canadian version of the International Classification of Diseases and Related Health Problems, 10th Revision (ICD‐10CA) and the Canadian Classification of Health Interventions (CCI).^19^ However, even that list was flawed in some respects; one shortcoming was the exclusion of severe pre‐eclampsia and HELLP syndrome cases due to coding limitations in the early version of ICD‐10 CA. This problem was identified by the CPSS in 2010^19^ and subsequently rectified by the Canadian Institute for Health Information; cases of severe pre‐eclampsia and HELLP syndrome can now be identified in Canadian hospitalisation data from 2012 onwards. Inclusion of conditions that did not necessarily represent SMM per se (eg asymptomatic HIV infection) and exclusion of some severe illnesses (eg surgical or manual correction of inverted uterus) were other limitations in the 2010 CPSS definition of composite SMM.

### 2.2 Definition of SMM

Severe maternal morbidity was defined as a set of heterogeneous maternal conditions known to be associated with severe illness and with prolonged hospitalisation or high case fatality. The definition was operationalised using an eclectic approach based on diagnostic, interventions and organ failure codes (see below).

### 2.3 List of SMM types and subtypes

The components of SMM were chosen through consensus by a multi‐disciplinary group of experts, who evaluated each candidate component in terms of feasibility of surveillance and validity (per the above‐mentioned definition of SMM). All SMM types and subtypes included in the 2010 list and others proposed for inclusion by the multi‐disciplinary group were evaluated in terms of frequencies, temporal trends, case fatality rates (ie death during the delivery admission), and (prolonged) length of hospital stay using hospitalisation data from Canada for the years 2006‐2015. The consensus assessments involved several meetings of the multi‐disciplinary group during which empirical data on each potential SMM type and subtype were reviewed, including the relevant code(s) in ICD‐10CA and CCI.

### 2.4 Data source

Information on these hospital deliveries (2006‐2015) was obtained from the Discharge Abstract Database of the Canadian Institute for Health Information, which contained records for approximately 98% of all deliveries in Canada (excluding Quebec). The database included information routinely abstracted from medical charts by trained personnel using standardised definitions and processes.^20^ Details regarding maternal and infant characteristics, labour and delivery events, and diagnoses and procedures were documented, with diagnoses coded using ICD‐10CA, and procedures coded using CCI. The validity of the information in the Discharge Abstract Database maternal and newborn records has been routinely assessed and shown to accurately reflect information contained in medical charts.^21^, ^22^

### 2.5 Components of SMM: SMM types and SMM subtypes

With SMM in any population defined as the frequency (incidence for new conditions, prevalence for pre‐existing ones) of heterogeneous maternal conditions known to be associated with severe illness, prolonged length of hospital stay, or high case fatality, we used diagnostic, intervention, and organ failure codes to identify eligible maternal diseases (eg eclampsia), interventions (eg hysterectomy), and conditions that signified organ failure (eg acute renal failure). Length of hospital stay was assessed using mean and median duration of hospital stay and the proportion of women with a prolonged length of hospital stay (≥7 days). Each candidate condition considered as signifying a potential SMM was evaluated by examining rates, temporal trends, length of stay, and case fatality rates for the years 2006‐2015 (with length of stay and case fatality contrasted among women with and without the candidate SMM). Once the SMM component (subtypes) list was finalised, the SMM subtypes were categorised for simplicity into SMM types based on aetiology, management, or other commonalities (eg the different forms of severe haemorrhage were grouped together, as were different surgical complications).

### 2.6 Descriptive epidemiology of SMM, Canada, 2012‐2016

This 2018 list of SMM conditions was then used to describe the epidemiology of SMM in Canada (excluding Quebec) for the years 2012‐2016. SMM rates were estimated as an overall composite, as well as broad SMM types, and individual SMM subtypes.

### 2.7 Statistical analysis

The frequencies of composite SMM, SMM types, and SMM subtypes were expressed using rates and 95% confidence intervals (CI). Case fatality rates and proportions of women with a prolonged hospital stay (≥7 days) were calculated similarly. Rates of composite SMM were estimated within categories of maternal age, parity, plurality, mode of delivery, and other factors, and contrasts between categories of a determinant were quantified using rate ratios and 95% confidence intervals. The population attributable fractions (PAF^23^) for prolonged hospital stay and maternal death associated with composite and specific SMM types/subtypes (or, in other words, the fraction of prolonged hospital stays and deaths that could be prevented by eliminating composite SMM or a specific SMM type/subtype) were estimated using the formula.

PAF=p∗(RR-1)/RR,

where PAF denotes the population attributable fraction, p denotes the proportion of cases with prolonged hospitalisation/death due to composite SMM or a specific SMM type/subtype, and RR denotes the rate ratio contrasting the rate of prolonged hospitalisation or death among women with composite SMM or with a specific SMM type/subtype vs women without SMM or without that specific SMM type/subtype. Analysis was carried out using SAS version 9.1 (SAS Institute).

### 2.8 Ethics considerations

Privacy considerations required the suppression of cells with small values (1‐4); in such cases, rates were provided as ranges calculated using 1 and 4 as the numerator. Since the study was based on de‐identified data (and conducted under the surveillance mandate of the Public Health Agency of Canada), no ethics approval from an Institutional Review Board was sought.

## 3 RESULTS

There were 2 843 395 hospital deliveries in Canada (excluding Quebec) between 2006 and 2015. Table 1 shows selected conditions assessed for inclusion as SMM subtypes. The assessment resulted in a deletion of some SMM components from the previous 2010 list (eg women with asymptomatic HIV infection) and the addition of new SMM components (eg acute fatty liver with plasma or red cell transfusion). Several candidate conditions assessed for inclusion were not added to the list (eg type 1 diabetes and obesity). The 2018 list of SMM subtypes (and their categorisation into broad SMM types) is presented in Table 2. Tables and Figures providing details regarding the various conditions evaluated as candidates for SMM are provided in Table S1.

**Table 1:** Frequency, length of hospitalisation, and case fatality rates associated with overall severe maternal morbidity (SMM), and for selected SMM types and subtypes evaluated for the new 2018 list of SMM, Canada (excluding Quebec), 2006‐2015 (based on 2 843 395 hospital deliveries)

Morbidity	Number	Rate per 100 000 deliveries	Case fatality rate*		Length of stay (days)	
			No. of deaths	Rate per 1000 deliveries	Mean	%≥7 d
No SMM (2010 list^16^)	2 801 128	98 513.5	12	0.004	2.3	1.1
At least one SMM (2010 list^16^)	42 267	1486.5	98	2.32	5.2	11.8
SMM on 2010 list; deleted from 2018 list						
HIV: asymptomatic infection or disease	1434	50.4	<5	0.70, 2.79	5.3	11.2
HIV: asymptomatic infection	1320	46.4	0	0.0	5.2	11.0
Hypertensive heart/renal disease	65	2.3	0	0.0	4.5	10.8
Evacuation of incisional haematoma	665	23.4	0	0.0	5.7	17.6
SMM on 2010 list; retained in 2018 list						
Acute renal failure without dialysis	844	29.7	9	10.7	10.6	45.1
Puerperal sepsis without ICU admission	1969	69.2	0	0.0	6.7	5.6
Evacuation incisional haematoma and RBC transfusion	133	4.7	0	0.0	7.5	33.1
HIV disease	114	4.0	<5	8.77, 35.1	5.8	13.2
Cardiomyopathy	605	21.3	<5	1.65, 6.61	6.5	33.2
New SMM evaluated; added to 2018 list						
Severe pre‐eclampsia^†^	2927	258.8	<5	0.34, 1.37	6.3	33.0
HELLP syndrome^†^	3124	276.2	<5	0.32, 1.28	5.3	22.4
Acute fatty liver^‡^ and plasma/RBC transfusion	236	8.3	5	21.2	9.7	33.5
Maternal intensive care unit admission	5454	191.8	57	10.5	9.0	32.4
Maternal intensive care unit admission ≤ 24 hours	2259	79.4	27	12.0	5.9	16.3
Inversion of uterus (vaginal delivery)	289	10.2	<5	3.46, 13.8	2.9	0.35
New SMM evaluated; not added to 2018 list						
Uterine rupture	2959	104.1	<5	0.34, 1.35	3.7	4.7
Acute fatty liver^‡^	12 505	439.8	6	0.48	3.1	3.5
Malignant neoplasms	1031	36.3	12	11.6	5.6	15.8
Thyroid disorders	21 034	739.7	<5	0.05, 0.19	3.2	4.8
Type 1 diabetes	2098	73.8	0	0.0	5.9	21.2
Type 2 diabetes	3220	113.2	<5	0.31, 1.24	4.2	12.8
Obesity	38 348	1348.7	<5	0.03, 0.10	3.1	4.7

Abbreviations: RBC, red blood cells; ICU, intensive care unit.

* If the numerator of the rate was >0 and <5, a range was provided (assuming a numerator of 1 and 4) instead of the actual value, and 95% interval estimates were not provided.

^†^ Based on hospitalisation data from 2012 to 2015.

^‡^ The conditions under acute fatty liver (O26.6) were expanded in ICD‐10‐CA version 2009 to include ‘Cholestasis (intrahepatic) in pregnancy’ and ‘Obstetric cholestasis’.

**Table 2:** Severe maternal morbidity (SMM) types, subtypes, and International Classification of Diseases (ICD‐10CA) and Canadian Classification of Health Interventions (CCI) codes for each SMM subtype

SMM type	SMM subtype	ICD‐10CA, CCI codes, and other variables
SPE, HELLP, eclampsia	Severe pre‐eclampsia, HELLP syndrome	O14.1, O14.2
	Eclampsia	O15
Severe haemorrhage	Placenta praevia with haemorrhage and red cell transfusion	O44.1 + RBCTRNSF = ‘Y’
	Placental abruption with coagulation defect	O45.0
	Antepartum haemorrhage with coagulation defect	O46.0
	Intrapartum haemorrhage with coagulation defect	O67.0
	Intrapartum haemorrhage with red cell transfusion	O67 + RBCTRNSF = ‘Y’
	Postpartum haemorrhage with red cell transfusion, procedures to the uterus, or hysterectomy	O72 + any of the following: RBCTRNSF = ‘Y’, or (1.RM.13, 1.KT.51, 5.PC.91.LA, or 5.PC.91.HV) + RBCTRNSF = 1, or (5.MD.60.RC, 5.MD.60.RD, 5.MD.60.KE, 5.MD.60.CB, or 1.RM.89.LA*), or 1.RM.87.LA‐GX
	Curettage with red cell transfusion	(5.PC.91.GA, 5.PC.91.GC, or 5.PC.91.GD) + RBCTRNSF = ‘Y’
Maternal ICU admission	Maternal ICU admission	FTSPCU in (‘10’,‘20’,‘25’,‘30’,‘35’,‘40’,‘45’,‘60’,‘80’)
Surgical complications	Complications of obstetric surgery and procedures	O75.4
	Evacuation of incisional haematoma with RBC transfusion	5.PC.73.JS + RBCTRNSF = ‘Y’
	Repair of bladder, urethra, or intestine	5.PC.80.JR, 1.NK.80, or 1.NM.80
	Reclosure of caesarean wound with RBC transfusion	(5.PC.80.JM or 5.PC.80.JH) + RBCTRNSF = ‘Y’
Hysterectomy	Caesarean hysterectomy	5.MD.60.RC, 5.MD.60.RD, 5.MD.60.KE, 5.MD.60.CB
	Hysterectomy using an open approach (without bladder neck suspension, suspension of vaginal vault, or pelvic floor repair)	1.RM.89.LA* (exclude if 1.PL.74, 1.RS.74, or 1.RS.80 code also present) or 1.RM.87.LA‐GX

Sepsis	Puerperal sepsis	O85
	Septicaemia during labour	O75.3
Embolism, shock, DIC	Obstetric shock	O75.1, R57, T80.5, or T88.6
	Obstetric embolism	O88
	Disseminated intravascular coagulation	D65
Assisted ventilation	Assisted ventilation through endotracheal tube	1.GZ.31.CA‐ND
	Assisted ventilation through tracheostomy	1.GZ.31.CR‐ND
	Cardiac complications of anaesthesia	O74.2, O89.1
Cardiac conditions	Cardiac complications of anaesthesia	O74.2, O89.1
	Cardiomyopathy	O90.3, I42, I43
	Cardiac arrest and resuscitation	I46, I49.0, 1.HZ.09, 1.HZ.30
	Myocardial infarction	I21, I22
	Pulmonary oedema and heart failure	I50, J81
Acute renal failure	Acute renal failure	O90.4, N17, N19 or N99.0
	Dialysis	1.PZ.21
Severe uterine rupture	Rupture of the uterus with red cell transfusion, procedures to the uterus, or hysterectomy	(O71.0 or O71.1) + any of the following: RBCTRNSF=‘Y’, or (1.RM.13, 1.KT.51, 5.PC.91.LA, or 5.PC.91.HV) + RBCTRNSF=‘Y’, or (5.MD.60.RC, 5.MD.60.RD, 5.MD.60.KE, 5.MD.60.CB, or 1.RM.89.LA*), or 1.RM.87.LA‐GX
Cerebrovascular accidents	Cerebral venous thrombosis in pregnancy	O22.5
	Cerebral venous thrombosis in the puerperium	O87.3
	Subarachnoid and intracranial haemorrhage, and cerebral infarction	I60, I61, I62, I63, or I64
	Acute fatty liver with red cell transfusion or plasma transfusion	O26.6 + (RBCTRNSF=‘Y’ or PLSTRNSF=’Y’)
	Hepatic failure	K71 or K72
	Cerebral oedema or coma	G93.6 or R40.2
Other types	Pulmonary, cardiac, and CNS complications of anaesthesia during pregnancy, labour, delivery, or the puerperium	O29.0, O29.1, O29.2, O89.0, O89.1, O89.2, O74.0, O74.1, O74.2, or O74.3
	Status asthmaticus	J45.01, J45.11, J45.81, or J45.91
	Adult respiratory distress syndrome	J80
	Acute abdomen	K35, K37, K65, N73.3, or N73.5
	Surgical or manual correction of inverted uterus for vaginal births only	5.PC.91.HQ or 5.PC.91.HP, restricted to vaginal births (ie absence of caesarean code 5.MD.60)
	Sickle cell anaemia with crisis	D57.0
	Acute psychosis	F53.1 or F23
	Status epilepticus	G41
	HIV disease	B20‐24, O98.7

Notes

*on selected diagnostic and procedure codes*:

• Canadian Institute for Health Information coding specific to severe pre‐eclampsia and HELLP (O14.1 and O14.2) began in 2012. The conditions under acute fatty liver (O26.6) were expanded in ICD‐10‐CA version 2009, to add codes for the sixth digits of ‘2’ (Delivered, with mention of postpartum complication) and ‘4’ (Postpartum condition or complication). Previously postpartum liver disorders may have been captured at O90.802 and O90.804 Other complications of the puerperium, not elsewhere classified, respectively. In addition, in ICD‐10‐CA version 2009 the conditions included in this code were expanded to include ‘Cholestasis (intrahepatic) in pregnancy’ and ‘Obstetric cholestasis’. Previously, cholestasis in pregnancy would have been classified as O99.6 Diseases of the digestive system complicating pregnancy, childbirth and the puerperium which included conditions in K80‐K93, and more specifically, K83.1 Cholestasis NEC.

• The CCI code 5.PC.91.HV Interventions to uterus (following delivery or abortion), compression using intrauterine balloon was introduced in CCI version 2012. Previously, this intervention may have been captured by code 5.PC.91.HT Interventions to uterus (following delivery or abortion) uterine (and vaginal) packing.

* 1.RM.89.LA is included only if codes 1.PL.74, 1.RS.74, or 1.RS.80 are NOT also present.

Table 3 shows the frequency, case fatality rate, and length of hospital stay associated with overall (composite) SMM for the period 2012 to 2016 based on the 2018 definition. Among the 1,418,545 deliveries during this period, the 22 799 cases of SMM yielded a composite SMM rate of 16.1 (95% CI 15.9, 16.3) per 1000 deliveries. Case fatality rates among women with and without SMM were 2.0 and 0.004 per 1000 deliveries, respectively. The median lengths of hospital stay were 4.0 and 2.0 days, respectively, among women with and without a severe maternal morbidity, while the corresponding proportions of women with a prolonged length of stay were 18.8% and 1.3%.

**Table 3:** Frequency, case fatality, and length of hospital stay (LOS) for composite severe maternal morbidity (SMM) and SMM types under the 2018 SMM definition, Canada (excluding Quebec), 2012‐2016

SMM type	Frequency		Case fatality		Length of stay	
	Number of cases	Rate per 100 000 (95% CI)	Number of deaths	Rate per 1000* (95% CI)	Median (days)	% with LOS ≥ 7 days
All deliveries	1 418 545	‐	51	0.04 (0.03, 0.05)	2.0	1.5
Any SMM	22 799	1607.2 (1586.6, 1628.0)	46	2.0 (1.5, 2.7)	4.0	18.8
No SMM	1 395 746	98 392.8 (98 3372.0, 98 413.4)	5	0.004 (0.001, 0.009)	2.0	1.3
Maternal ICU admission	2729	192.4 (185.2, 199.8)	24	8.8 (5.9, 13.1)	5.0	36.9
Severe pre‐eclampsia, HELLP, eclampsia	7923	558.5 (546.4, 570.9)	<5	0.13, 0.50*	5.0	26.2
Severe haemorrhage	7085	499.5 (487.9, 511.2)	19	2.7 (1.7, 4.2)	3.0	14.2
Severe uterine rupture	204	14.4 (12.5, 16.5)	<5	4.9, 19.6*	5.0	27.5
Hysterectomy	2109	148.7 (142.4, 155.2)	6	2.8 (1.3, 6.1)	3.0	22.6
Sepsis	1296	91.4 (86.5, 96.5)	<5	0.77, 3.09*	4.0	26.1
Embolism, shock, or DIC	973	68.6 (64.3, 73.0)	19	19.5 (12.5, 30.3)	4.0	28.2
ARF or dialysis	647	45.6 (42.2, 49.3)	9	13.9 (7.3, 26.2)	7.0	53.0
Cardiac conditions	887	62.5 (58.5, 66.8)	36	40.6 (29.5, 55.7)	5.0	34.9
Cerebrovascular accidents	136	9.6 (8.1, 11.4)	5	36.8 (15.8, 83.2)	3.0	27.9
Surgical complications	2752	194.0 (186.8, 201.4)	18	6.5 (4.1, 10.3)	3.0	13.1
Assisted ventilation	940	66.3 (62.1, 70.6)	29	30.9 (21.6, 44.0)	6.0	47.8

Abbreviations: CI, confidence interval; ICU, intensive care unit; DIC, disseminated intravascular coagulation; ARF, acute renal failure.

* If the numerator of the rate was >0 and <5, a range was provided (assuming a numerator of 1 and 4) instead of actual value (95% confidence interval not provided).

Rates of composite SMM were significantly higher among women aged 15‐19 years, 35‐39 years, and ≥40 years, being 18.8, 19.2, and 30.2 per 1000 deliveries, respectively, compared with 15.0 per 1000 deliveries among women aged 20‐24 years Table 4. SMM rates were also significantly higher among nulliparous women,women with increasing parity, multi‐fetal pregnancy, or previous caesarean delivery; and among women with labour induction or caesarean delivery. Women who received epidural anaesthesia had lower rates of composite SMM than those who did not (12.5 vs 19.0 per 1000 deliveries).

**Table 4:** Numbers and rates of women with severe maternal morbidity by maternal and clinical characteristics, Canada (excluding Quebec), 2012‐2016

Maternal characteristics	Number of deliveries	Severe maternal morbidity		
		Number	Rate per 10 000 deliveries (95% CI)	Rate Ratio (95% CI)
Age (years)				
<15	307	5	162.9 (53.1, 376.0)	1.09 (0.46, 2.60)
15‐19	43 862	824	187.9 (175.4, 201.0)	1.25 (1.16, 1.35)
20‐24	184 064	2757	149.8 (144.3, 155.4)	1.00 (Reference)
25‐29	403 695	5592	138.5 (134.9, 142.2)	0.92 (0.88, 0.97)
30‐34	491 668	7395	150.4 (147.0, 153.8)	1.00 (0.96, 1.05)
35‐39	242 526	4644	191.5 (186.1, 197.0)	1.28 (1.22, 1.34)
≥40	52 423	1582	301.8 (287.3, 316.8)	2.01 (1.90, 2.14)
Parity				
0	491 931	9819	199.6 (195.7, 203.6)	1.67 (1.62, 1.73)
1	388 503	4636	119.3 (115.9, 122.8)	1.00 (Reference)
2	154 894	2112	136.4 (130.6, 142.3)	1.14 (1.09, 1.20)
3	54 132	845	156.1 (145.8, 166.9)	1.31 (1.22, 1.41)
4	19 904	355	178.4 (160.4, 197.7)	1.49 (1.34, 1.66)
≥5	18 252	366	200.5 (180.7, 221.9)	1.68 (1.51, 1.87)
Missing	290 929	4666	160.4 (155.8, 165.0)	1.34 (1.29, 1.40)
Elderly primigravida				
Yes	17 189	577	335.7 (309.3, 363.7)	2.12 (1.95, 2.30)
No	1 401 356	22 222	158.6 (156.5, 160.7)	1.00 (Reference)
Previous caesarean delivery				
Yes	199 829	4264	213.4 (207.1, 219.8)	1.40 (1.36, 1.45)
No	1 218 716	18 535	152.1 (149.9, 154.3)	1.00 (Reference)
Epidural anaesthesia				
Yes	640 186	8029	125.4 (122.7, 128.2)	0.66 (0.64, 0.68)
No	778 359	14 770	189.8 (186.7, 192.8)	1.00 (Reference)
Labour induction				
Yes	370 175	7714	208.4 (203.8, 213.0)	1.45 (1.41, 1.49)
No	1 048 370	15 085	143.9 (141.6, 146.2)	1.00 (Reference)
Caesarean delivery				
Yes	404 319	13 744	339.9 (334.4, 345.6)	3.81 (3.71, 3.91)
No	1 014 226	9055	89.3 (87.5, 91.1)	1.00 (Reference)
Plurality				
Singleton	1 394 775	21 402	153.4 (151.4, 155.5)	1.00 (Reference)
Twin	23 326	1348	577.9 (548.3, 608.6)	3.77 (3.57, 3.97)
Triplet or higher‐order	443	48	1083.5 (809.8, 1410.8)	7.06 (5.40, 9.23)
Total	1 418 545	22 799	16.1 (15.9, 16.3)	‐

The frequency, case fatality rates, and length of stay for broad types of SMM are shown in Table 3 (SMM types are not mutually exclusive). The most common types of SMM included severe pre‐eclampsia, eclampsia, and HELLP syndrome,severe haemorrhage; surgical complications; maternal intensive care unit admission; and hysterectomy. Table 5 provides the same details for each SMM subtype (SMM subtypes are not mutually exclusive). Severe pre‐eclampsia and HELLP syndrome (514.6 per 100 000 deliveries), postpartum haemorrhage with red cell transfusion, procedures to the uterus or hysterectomy (433.2 per 100 000), maternal intensive care unit admission (192.4 per 100 000), hysterectomy (148.7 per 100 000), and complications of surgery and procedures (106.9 per 100 000) were the most common SMM subtypes.

**Table 5:** Severe maternal morbidity (SMM) subtypes and associated case fatality and length of hospital stay (LOS), Canada (excluding Quebec), 2012‐2016 (based on 1 418 545 hospital deliveries)

SMM subtypes	Number of SMM cases	Rate per 100 000 (95% CI)	Case fatality rate/1000	Median LOS (days)	% with LOS ≥ 7 d*
Severe pre‐eclampsia or HELLP syndrome	7300	514.6 (502.9, 526.5)	0.14, 0.55*	5.0	26.9
Eclampsia	668	47.1 (43.5, 50.8)	0.0	4.0	19.5
Cerebral venous thrombosis in pregnancy	35	2.5 (1.7, 3.5)	0.0	2.0	2.8, 11.4*
Cerebral venous thrombosis in the puerperium	6	0.4 (0.1, 0.9)	0.0	20.5	16.7, 66.7*
Cerebrovascular accidents–stroke	98	6.9 (5.6, 8.4)	51.0	4.0	36.7
Placenta praevia with haemorrhage and RBC transfusion	531	37.4 (34.3, 40.7)	1.9, 7.5*	5.0	44.6
Placental abruption with coagulation defect	275	19.4 (17.2, 21.8)	3.6, 14.5*	3.0	16.7
Antepartum haemorrhage with coagulation defect	79	5.6 (4.4, 7.0)	12.7, 50.6*	3.0	16.5
Intrapartum haemorrhage with coagulation defect	107	7.5 (6.2, 9.1)	9.3, 37.4*	4.0	21.5
Intrapartum haemorrhage with RBC transfusion	352	24.8 (22.2, 27.5)	2.8, 11.4*	4.0	19.3
Postpartum haemorrhage with RBC transfusion, procedures to the uterus, or hysterectomy	6145	433.2 (422.4, 444.2)	2.4	3.0	13.1
Curettage with RBC transfusion	934	65.8 (61.7, 70.2)	1.1, 4.3*	3.0	7.1
Rupture of the uterus with RBC transfusion or procedures to the uterus or hysterectomy	204	14.4 (12.5, 16.5)	4.9, 19.6*	5.0	27.5
Cardiac conditions	887	62.5 (58.5, 66.8)	40.6	5.0	34.9
Cardiac complications of anaesthesia	56	3.9 (3.0, 5.1)	17.9, 71.4*	3.0	1.8, 7.1*
Cardiomyopathy	352	24.8 (22.2, 27.5)	2.8, 11.4*	3.0	26.4
Cardiac arrest and resuscitation	141	9.9 (8.3, 11.7)	241.1	4.0	32.0
Myocardial infarction	13	0.9 (0.5, 1.5)	0	6.0	38.5
Pulmonary oedema and heart failure	410	28.9 (26.2, 31.8)	2.4, 9.8*	9.0	49.8
Septicaemia during labour	302	21.3 (18.9, 23.8)	3.3, 13.2*	3.0	12.9
Puerperal sepsis	997	70.3 (66.0, 74.8)	1.0, 4.0*	5.0	30.1
Obstetric shock	486	34.3 (31.3, 37.5)	28.8	4.0	30.3
Obstetric embolism	422	29.7 (27.0, 32.8)	16.6	4.0	24.4

SMM subtypes	Number of SMM cases	Rate per 100 000 (95% CI)	Case fatality rate/1000*	Median LOS (days)	% with LOS ≥ 7 d*
Disseminated intravascular coagulation	113	8.0 (6.6, 9.5)	8.8, 35.4*	6.0	47.8
Acute renal failure	620	43.7 (40.3, 47.3)	14.5	7.0	53.1
Dialysis	74	5.2 (4.1, 6.5)	67.6	14.5	77.0
Hysterectomy	2109	148.7 (142.4, 155.2)	2.8	3.0	22.6
HIV disease	272	19.2 (17.0, 21.6)	0.0	3.3	2.0
Complications of obstetric surgery and procedures	1516	106.9 (101.6, 112.3)	11.9	3.0	15.1
Evacuation of incisional haematoma with RBCT	56	3.9 (3.0, 5.1)	0.0	6.0	44.6
Repair of bladder, urethra, or intestine	1044	73.6 (69.2, 78.2)	0.0	3.0	8.3
Reclosure of caesarean wound with RBCT transfusion	187	13.2 (11.4, 15.2)	0.0	4.0	22.5
Maternal intensive care unit admission	2729	192.4 (185.2, 199.8)	8.8	5.0	36.9
Acute fatty liver with RBCT or plasma transfusion	148	10.4 (8.9, 12.3)	6.8, 27.0*	5.0	39.9
Pulmonary, cardiac, and CNS complications of anaesthesia during pregnancy/delivery/puerperium	159	11.2 (9.6, 13.1)	6.3, 25.2*	3.0	6.3
Surgical or manual correction of inverted uterus	135	9.5 (8.0, 11.3)	7.4, 29.6*	2.0	0.7, 3.0*
Status asthmaticus	24	1.7 (1.1, 2.5)	41.7, 166.7*	2.0	4.2, 16.7*
Adult respiratory distress syndrome	56	3.9 (3.0, 5.1)	17.9, 71.4*	7.5	51.8
Acute abdomen	117	8.2 (6.8, 9.9)	8.5, 34.2*	5.0	36.8
Hepatic failure	34	2.4 (1.7, 3.3)	147.1	8.0	55.9
Assisted ventilation through endotracheal tube	928	65.4 (61.3, 69.8)	31.3	6.0	47.6
Assisted ventilation through tracheostomy	19	1.3 (0.8, 2.1)	0.0	27.0	68.4
Sickle cell anaemia with crisis	56	3.9 (3.0, 5.1)	0.0	7.0	55.4
Acute psychosis	43	3.0 (2.2, 4.1)	0.0	4.0	23.3
Status epilepticus	48	3.4 (2.5, 4.5)	0.0	4.5	27.1
Cerebral oedema or coma	13	0.9 (0.5, 1.5)	76.9, 307.7*	3.0	7.7, 30.8*

Abbreviations: CI, confidence interval; CNS, central nervous system; RBCT, red blood cell transfusion.

* If numerator of the rate was >0 and <5, a range was provided (assuming a numerator of 1 and 4) instead of the actual value.

Case fatality rates were highest among women with cardiac arrest and resuscitation (241.1 per 1000), hepatic failure (147.1 per 1000), and dialysis (67.6 per 1000), and among those with cerebrovascular accidents (51.0 per 1000). Women with several different SMM subtypes had an extended hospital stay, with ≥40% having a hospital stay ≥7 days among those with placenta praevia requiring blood transfusion, pulmonary oedema and heart failure, disseminated intravascular coagulation, acute renal failure/dialysis, evacuation of incisional haematoma requiring transfusion, acute fatty liver requiring transfusion, assisted ventilation, or sickle cell anaemia with crisis Table 5.

Rate ratios and PAFs for each SMM type are presented in Table 6. The rate ratio for a prolonged hospital stay among women with any SMM (vs those without) was 14.5, while the rate ratio for prolonged hospital stay among women admitted to an ICU (vs those not admitted to an ICU) was 24.4. The rate ratio for maternal death among women with any severe maternal morbidity (vs those without) was 459.1, and among those admitted to ICU (vs those not admitted to ICU), the rate ratio for death was 461.7. The PAF for maternal death associated with any SMM was 88.0% (95% CI 74.6, 94.4), while that associated with maternal ICU admission was 47.0% (95% CI 31.3, 59.1). Thus, preventing all SMM cases (per the 2018 SMM definition) would eliminate 88% of maternal deaths, while preventing SMM resulting in maternal ICU admission would eliminate 47% of maternal deaths. The PAF for prolonged hospital stay associated with any SMM was 17.8% (95% CI 17.3, 18.3), while the PAF for prolonged hospital stay associated with maternal ICU admission was 4.3% (95% CI 4.0, 4.6).

**Table 6:** Frequencies of specific severe maternal morbidity (SMM) types, and associated rate ratios and population attributable fractions (PAF) for case fatality and prolonged length of hospital stay (LOS), Canada (excluding Quebec), 2012‐2016

Morbidity	Prolonged LOS		Case fatality	
	Rate ratio (95% CI)	PAF (95% CI)	Rate ratio (95% CI)	PAF (95% CI)
Any severe maternal morbidity	14.5 (14.0, 14.9)	17.8 (17.3, 18.3)	459.1 (195.9, 1076.0)	88.0 (74.6, 94.4)
Severe pre‐eclampsia, HELLP, eclampsia	18.1 (17.5, 18.9)	8.75 (8.36, 9.13)	3.6, 15.2*	1.8, 5.4*
Cerebrovascular accident	17.7 (13.5, 23.2)	0.16 (0.11, 0.20)	1133.6 (457.5, 2809.3)	9.8 (1.3, 17.6)
Severe haemorrhage	9.36 (8.82, 9.92)	4.01 (3.73, 4.28)	118.3 (67.1, 208.6)	37.2 (22.1, 49.0)
Severe uterine rupture	17.4 (13.9, 21.8)	0.24 (0.17, 0.30)	139.1, 591.7*	2.0, 7.8*
Cardiac conditions	22.4 (20.5, 24.5)	1.32 (1.17, 1.47)	3835.8 (2107.8, 6980.4)	70.6 (55.0, 80.8)
Sepsis	16.7 (15.3 18.4)	1.42 (1.26, 1.58)	21.9, 93.1*	1.9, 7.8*
Obstetric embolism, shock, DIC	18.0 (16.3, 19.9)	1.15 (1.01, 1.30)	865.0 (492.1, 1520.7)	37.2 (22.4, 49.2)
Acute renal failure/dialysis	34.0 (31.6, 36.6)	1.48 (1.32, 1.65)	469.6 (229.5, 960.7)	17.6 (6.44, 27.4)
Hysterectomy	14.6 (13.5, 15.8)	1.98 (1.79, 2.17)	89.5 (38.2, 209.7)	11.4 (2.32, 20.1)
Surgical complications	8.42 (7.64, 9.27)	1.42 (1.25, 1.58)	280.6 (158.2, 497.7)	34.9 (20.6, 47.6)
Maternal ICU admission	24.4 (23.2, 25.7)	4.31 (4.03, 4.58)	461.2 (266.4, 798.2)	47.0 (31.3, 59.1)
Assisted ventilation	30.8 (28.8, 33.0)	1.94 (1.75, 2.12)	1987.9 (1146.4, 3447.1)	56.9 (40.8, 68.5)

Notes

Rate ratios contrast the rate of prolonged hospitalisation/death among women with any severe morbidity vs women without severe morbidity (and the presence of specific SMM types with the absence of that SMM type). The population attributable fraction expresses the fraction of women with a prolonged hospital stay/death that could be eliminated by preventing all SMM or by preventing a specific SMM type. Note: SMM types are not mutually exclusive.

Abbreviation: DIC, disseminated intravascular coagulation.

* If the numerator of the rate was >0 and <5, a numerator of 1 and 4 was used to calculate the rate and 2 rate ratios and PAFs were estimated (95% CI not provided).

## 4 COMMENT

### 4.1 Principal findings

We used a priori knowledge and empirical support from frequencies, temporal trends, case fatality rates, and length of hospital stay to derive a list of conditions for SMM surveillance. The rate of composite SMM according to the revised list was 16.1 per 1000 deliveries in Canada, 2012‐2016. This rate was substantially higher among older women, primiparous women, women with high parity, multi‐fetal pregnancy, previous caesarean delivery, and women who had labour induction or a caesarean delivery. The most common SMM subtypes were severe pre‐eclampsia and HELLP syndrome, severe postpartum haemorrhage (ie postpartum haemorrhage requiring red cell transfusion, procedures to the uterus or hysterectomy), maternal intensive care unit admission, hysterectomy, and complications of surgery and procedures. Case fatality rates were highest among women with cardiac arrest and resuscitation, hepatic failure, those receiving dialysis, those with cerebrovascular accidents, and those with cardiac conditions. SMM was associated with a PAF of 47% to 18 %for prolonged hospitalisation and a PAF of 88% for maternal death.

### 4.2 Strengths of the study

The strengths of our study and the proposed SMM surveillance framework include reliance on multi‐disciplinary input and evidentiary support from contemporary data on deliveries. The hospitalisation data source (viz., the Discharge Abstract Database of the Canadian Institute for Health Information) has made changes in ICD‐10CA coding (eg introduction of a code for severe pre‐eclampsia in 2012) and other important aspects of data collection (linked mother and infant records, extraction of gestational age at delivery, etc), which have facilitated comprehensive monitoring of SMM.

### 4.3 Limitations of the data

Limitations of our study include an inability to identify some clinically relevant cases based on ICD‐10CA codes (eg extreme obesity) and to distinguish between some pre‐existing and acute complications arising in pregnancy. We were unable to identify women who received more than one transfusion (a measure of more severe morbidity than haemorrhage with any transfusion), as our data source documented the type of blood component transfused but not the number of units. Collection of information on the number of pints of blood transfused would enable more accurate specification of haemorrhage severity and organ failure, and improved maternal health surveillance.

Although overall SMM had a PAF of 88% for maternal death, the sum of the PAFs of the 12 individual SMM types exceeded 100%. This was expected, as the SMM types overlapped, both as illness entities and potentially as component causes of a sufficient cause(s) model of maternal death (or prolonged hospitalisation). Finally, our inability to include information from the province of Quebec (which did not contribute to the Discharge Abstract Database) was another limitation of our study.

### 4.4 Interpretation

Conditions included as components of severe maternal morbidity vary considerably in the literature.^3^, ^4^, ^5^, ^6^, ^7^, ^24^, ^25^, ^26^, ^27^ For instance, Euro‐Peristat (a part of the European Union's Health Monitoring Program) defined SMM as a composite of eclampsia, hysterectomy for postpartum haemorrhage, ICU admission, blood transfusion, or uterine artery embolisation.^24^ On the other hand, the EPIMOMS study group in France defined severe maternal morbidity to include the EURO‐PERISTAT indicators, as well as measures of organ system dysfunction (a total of 17 items).^25^ Such differences are at least partly due to a failure to formally define SMM per se (ie without reference to its component conditions) and instead rely on a selected list of SMM conditions chosen without specification as to criteria for inclusion and exclusion of candidate conditions. Our definition of SMM, which was based on a priori knowledge of illness severity and empirical assessments of prolonged hospitalisation and case fatality, ensured a high PAF for maternal death. More restrictive definitions, such as the EURO‐PERISTAT definition, which are focussed on direct obstetric morbidity, may not capture SMM due to cardiac conditions, surgical morbidity, and other complications which contribute substantially to prolonged hospitalisation and case fatality (Table 3).

Published studies of population rates of SMM fall into three primary types, which differ based on the conceptual framework used for SMM surveillance. The EURO‐PERISTAT framework is based on the premise that surveillance of SMM can be restricted to key conditions that are feasible to assess in a valid manner. On the other hand, the WHO proposal for surveillance of maternal near‐miss (ie severe acute maternal morbidity) recommends the prospective follow‐up of severe maternal complications (ie potentially life‐threatening conditions) with a view to accurately and comprehensively identifying cases of organ system failure.^28^, ^29^ Some studies attempting to use the latter framework for near‐miss surveillance have documented underestimation of rates of SMM and maternal death both in high‐income and in low‐income settings.^30^, ^31^ This is partly because such studies^30^, ^31^ have typically employed retrospective assessments of organ system failure. However, prospective follow‐up of all potentially life‐threatening conditions is challenging even in high‐income settings. Our study describes the third type of SMM surveillance framework previously used by countries such as Australia, Canada, England, and the United States.^5^, ^6^, ^7^, ^17^, ^18^, ^19^, ^25^, ^27^, ^32^ This third framework employs a retrospective design and an eclectic approach (using disease‐based, intervention‐based, and organ system failure‐based criteria) to identify cases of SMM in routine hospitalisation data that include diagnosis, intervention codes, and other information.

The list of SMM types and subtypes used in our study was similar, although perhaps more comprehensive than the maternal morbidity outcome indicator created by Roberts et al who identified ‘true’ severe maternal morbidity by examining the medical records of 400 cases of suspected severe morbidity and 800 non‐cases.^32^ In the Roberts et al study, three clinicians reviewed the medical records, identified SMM based on a clinical gestalt, and created a list of 11 morbid conditions and 15 procedures. We defined SMM to include severe maternal illnesses associated with prolonged length of hospital stay or high case fatality. Thus, conditions such as severe pre‐eclampsia, HELLP syndrome, and eclampsia constituted an SMM, since 26% of women with these conditions had a prolonged LOS. Conversely, 74% of such women did not have a prolonged LOS, but this was not a repudiation of the definition. Also, women with such severe illnesses and a LOS <  7 days were included as cases of SMM as it is possible to be severely ill, receive life‐saving intervention, and recover fairly quickly.

We faced some challenges, however, in translating our SMM definition into an operational list of severely morbid conditions. One limitation arose from our reliance on ICD‐10CA codes, which do not capture all conditions of interest with sufficient accuracy. Extreme obesity, a maternal condition which could potentially satisfy our definition of SMM, is an example: ICD‐10CA includes a code for obesity but not extreme obesity. Additionally, obesity, which did not satisfy the SMM definition in terms of case fatality rates and prolonged length of stay, was captured in only a small fraction of women: less than 2% vs an expected frequency of over 10%.^10^, ^33^ We omitted conditions such as malignancy in pregnancy, which were associated with prolonged length of stay and high case fatality, from the SMM list because the associated burden of illness appeared to be mostly unrelated to pregnancy, and we were unable to identify cases in which the course of the malignancy was aggravated by pregnancy. On the other hand, we included conditions such as maternal ICU admission, which did not identify any additional deaths but did carry a high risk of prolonged length of stay.^34^ Finally, we encountered a few conditions (eg diabetes mellitus, asymptomatic HIV infection) where the prolonged length of hospital stay was likely associated with management or socio‐economic issues rather than severe morbidity per se.

The rate ratios and PAFs associated with specific types of SMM in our study can be used to inform clinical practice and public health policy. From a public health standpoint, PAFs for maternal death show that cardiac conditions, haemorrhage, obstetric embolism, obstetric shock, DIC, and surgical complications are the priorities to be addressed in terms of SMM prevention. A substantial reduction in maternal mortality would likely result from a reduction in these SMM. Adverse temporal trends or geographic differences identified in our study also provide an impetus for action, whether nationally or at the provincial level. Audit of maternal deaths and SMM cases is a worthwhile undertaking that is being increasingly discussed in clinical circles, and such activities could help focus attention on prevention of SMM and maternal death through improved care.

### 4.5 Conclusions

We combined a priori clinical knowledge, prolonged length of stay, high case fatality, and expert consensus to identify a set of severely morbid conditions that could enable robust surveillance of SMM in Canada and other high‐income countries. These SMM subtypes, which vary in frequency, case fatality, and PAFs for maternal death, suggest that significant reductions in maternal mortality will result from the prevention or improved care of SMM, especially cardiac conditions, severe haemorrhage, obstetric embolism, obstetric shock, DIC, and surgical complications. Our 2018 list of SMM types and subtypes should help improve surveillance and benchmarking of SMM in Canada and elsewhere.
