# Supplementary material for: Severe maternal morbidity surveillance: Monitoring pregnant women at high risk for prolonged hospitalisation and death
Source: Paediatr Perinat Epidemiol. 2019 Aug 12;34(4):427–39. doi: 10.1111/ppe.12574 (PMC7383693; doi:10.1111/ppe.12574)
Supplement: Supplementary file 1 [file PPE-34-427-s001.doc]

**eTable 1**

Note: Evaluation of each candidate SMM in terms of prolonged length of hospital stay and case fatality was based on a comparison with the same rates among women with SMM and women without SMM (with SMM defined using the 2010 definition^16^). The latter rates appear in each Table.

Analyses carried out to ascertain if women with specific diagnoses and/or receiving specific interventions were to be considered cases of severe maternal morbidity (SMM).

**Table of Contents Page**

1. Acute renal failure…………………………….………………….……………...............2
2. Puerperal sepsis………………………………………………………………………….4
3. Maternal intensive care unit admission…………………..….…….…………………….6
4. Maternal intensive care unit admission <24 hours vs >=24 hours……………………....8
5. Uterine rupture …….………………………………………………………………… ....9
6. Procedures to control postpartum hemorrhage………………………………….……....11
7. Red cell transfusion……………………………………………………………………..13
8. Hysterectomy…………………………………………………………………………....14
9. Acute fatty liver………………………………………………………………….……...16
10. Severe pre-eclampsia and HELLP syndrome………………………………….…….….18
11. Cardiac conditions………………………………………………………………………20
12. Human immunodeficiency virus infection………………………………………….…..24
13. Re-closure of caesarean wound……………………………………….………….....…..26
14. Inversion of uterus……………………………………………………………….……...27
15. Uterine curettage………………………………………………………………………...28
16. Pre-existing hypertensive heart/renal disease……………………………..…………….30
17. Cardiomyopathy………………………………………………………………..………..31
18. Miscellaneous conditions……………………………………………………..…..……. 32

_____________________________________________________________________________

**Issue 1.** Should all cases of acute renal failure be included as cases of severe maternal morbidity (SMM) or should such cases be restricted to those requiring dialysis?

**Results:** The frequency of acute renal failure ranged between 2.8 and 5.3 per 10,000 deliveries between 2006 and 2015. Cases without associated dialysis were more frequent and there was an increasing trend among cases not associated with a dialysis code (Figure 1a). The case fatality rates were substantial among both cases of acute renal failure associated with a dialysis code and those not associated with a dialysis code. Similarly, length of hospital stay was prolonged both among those with and without associated dialysis codes.

**Decision:** Retain all cases of acute renal failure as a component of severe maternal morbidity.

Table 1. Case fatality rates among women with acute renal failure who did and did not have an associated code for dialysis, Canada (excluding Quebec), 2006-2015.

| Morbidity status | Case fatality rate (CFR) | | |  | Length of stay | | |
| --- | --- | --- | --- | --- | --- | --- | --- |
|  | Deaths | Total deliveries | CFR (per 10,000 deliveries) |  | Mean  (All cases) | Mean  (excl. deaths) | % >7days  (all cases) |
| No SMM | 12 | 2,801,128 | 0.04 |  | 2.3 | 2.3 | 1.1 |
| At least one SMM | 98 | 42,267 | 23.2 |  | 5.2 | 5.2 | 11.8 |
| Acute Renal Failure (all) | 18 | 912 | 197.4 |  | 11.8 | 11.7 | 48.6 |
| With Dialysis | 9 | 68 | 1,323.5 |  | 26.3 | 26.7 | 91.2 |
| Without Dialysis | 9 | 844 | 106.6 |  | 10.6 | 10.6 | 45.1 |

SMM denotes severe maternal morbidity as previously defined by the Canadian Perinatal Surveillance System.^16^


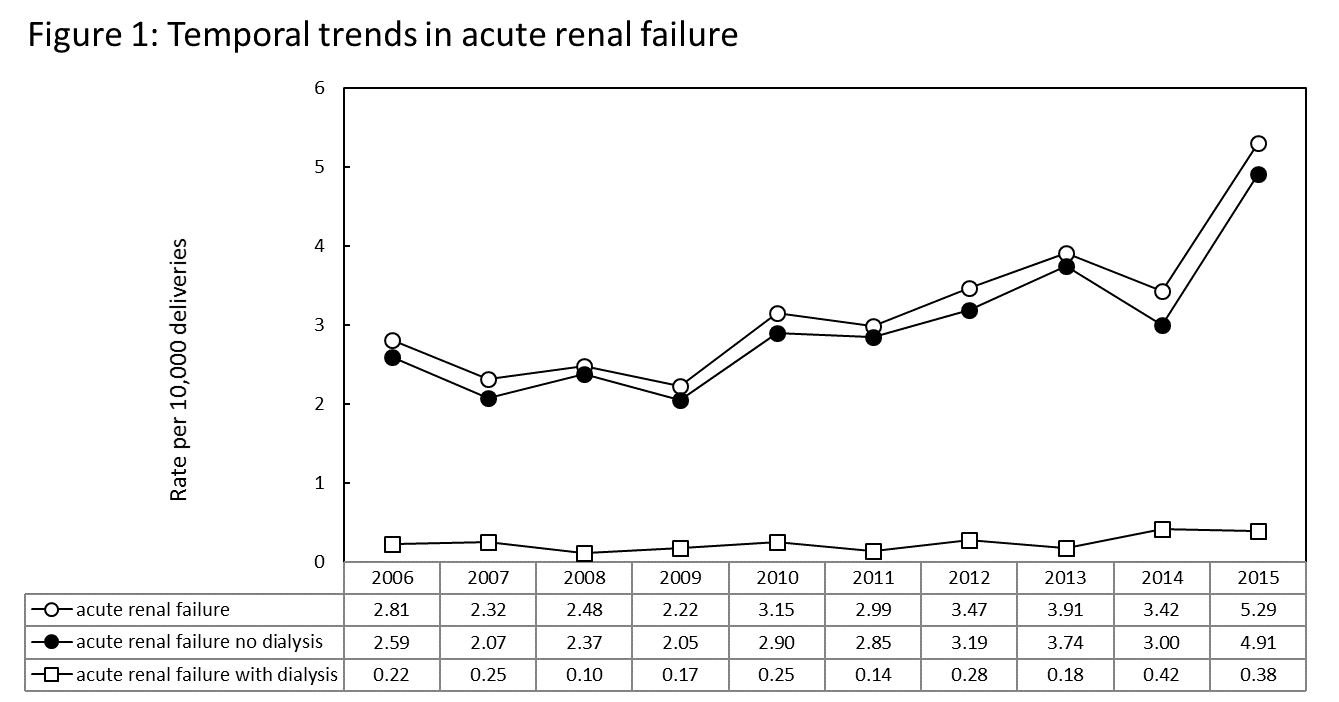


**Issue 2.** Should all cases of puerperal sepsis be included as cases of severe maternal morbidity or should such cases be restricted to cases of puerperal sepsis requiring ICU admission?

**Results:** Rates of puerperal sepsis ranged between 8.8 and 6.7 per 10,000 deliveries between 2006 and 2015 and a decreasing trend was evident especially among cases not admitted to ICU (Figure 1b). Sepsis cases admitted not to ICU were more frequent than those admitted to ICU. There were no deaths among women with sepsis who were not admitted to ICU, while death rates among sepsis cases in ICU were high (Table 1). Hospital stay was prolonged among sepsis cases irrespective of whether they were admitted to ICU.
**Decision:** Retain all cases of sepsis as a component of severe maternal morbidity.

Table 2. Case fatality rates among women with peurperal sepsis who were and were not admitted to an intensive care unit (ICU), Canada (excluding Quebec), 2006-2015.

| Morbidity status | Case fatality rate (CFR) | | |  | Length of stay | | |
| --- | --- | --- | --- | --- | --- | --- | --- |
|  | Deaths | Total deliveries | CFR (per 10,000 deliveries) |  | Mean  (All cases) | Mean  (excl. deaths) | % > 7days  (all cases) |
| No SMM | 12 | 2,801,128 | 0.04 |  | 2.3 | 2.3 | 1.1 |
| At least one SMM | 98 | 42,267 | 23.2 |  | 5.2 | 5.2 | 11.8 |
| Puerperal Sepsis (all) | 6 | 2,155 | 27.8 |  | 7.6 | 7.6 | 4.8 |
| No ICU | 0 | 1,969 | 0.0 |  | 6.7 | 6.7 | 5.6 |
| With ICU | 6 | 186 | 322.6 |  | 17.6 | 17.7 | 8.9 |

SMM denotes severe maternal morbidity as previously defined by the Canadian Perinatal Surveillance System.^16^


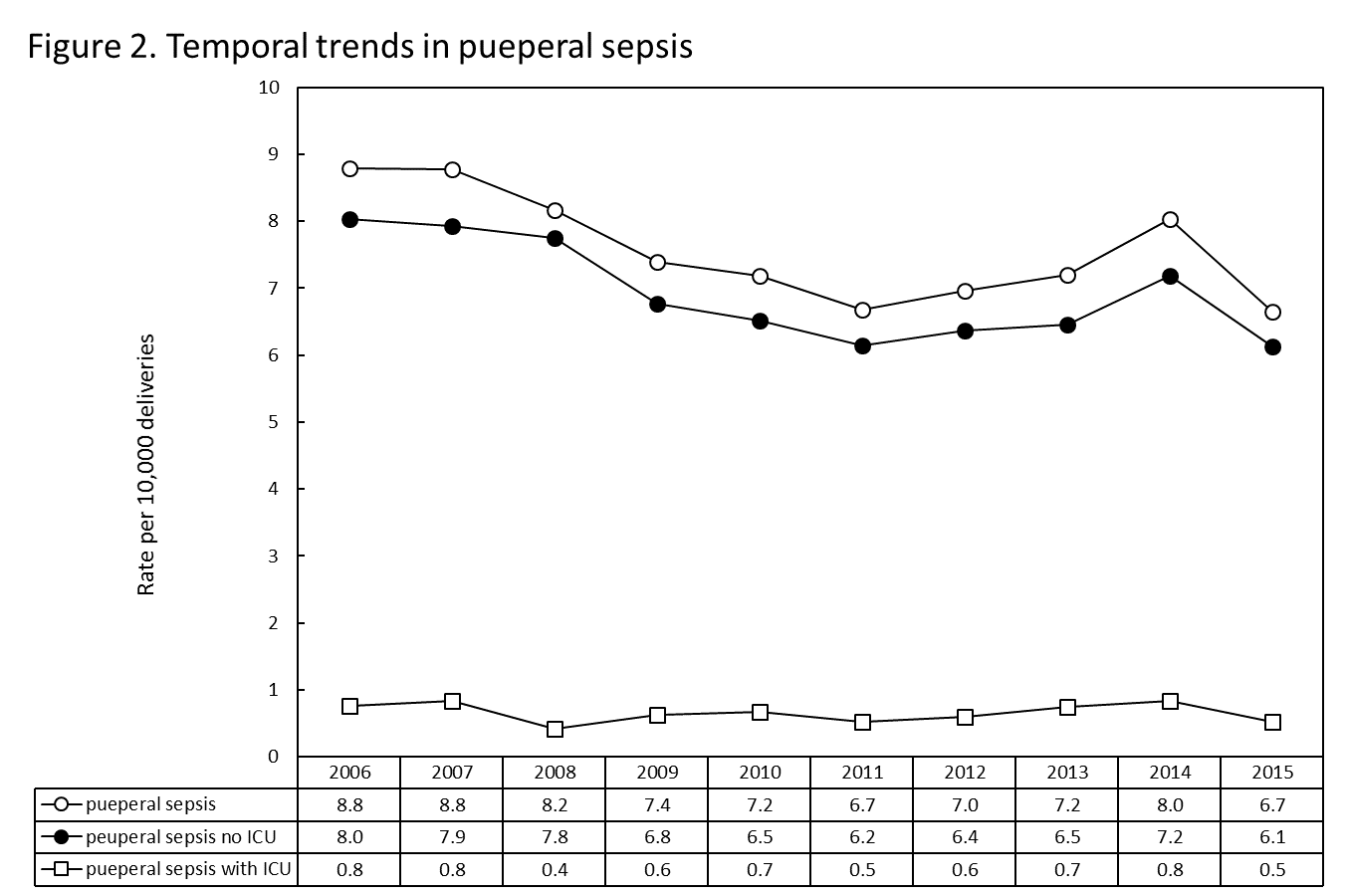


**Issue 3.** Should women admitted to an intensive care unit (ICU) be considered cases of severe maternal morbidity?

**Results:** Case fatality rates show that inclusion of ICU admissions as an SMM does not increase identification of women who die, as all such women had at least one of the SMM diagnostic or procedure codes. However, the length of stay associated with ICU admission without an SMM was longer (mean 6.4, 20.8% with 7+ days) than the length of stay associated with at least one SMM (mean 5.2 days, 11.8% with 7+ days).

**Decision:** Consider including maternal ICU admission as a severe maternal morbidity.

**Table 3. Frequency, case fatality rates and length of stay among maternal intensive care unit (ICU) admissions, Canada (excluding Quebec) 2006-2015.**

| Morbidity status | Case Fatality Rate | | |  | Length of stay (days) | | |
| --- | --- | --- | --- | --- | --- | --- | --- |
|  | Deaths | Total Cases | CFR/ 10,000 deliveries |  | Mean  (all cases) | Mean  (excl deaths) | % >7 days  (all cases) |
| No SMM | 12 | 2,801,128 | 0.04 |  | 2.3 | 2.3 | 1.1 |
| At least one SMM | 98 | 42,267 | 23.2 |  | 5.2 | 5.2 | 11.8 |
| No ICU admission | 53 | 2,837,941 | 0.2 |  | 2.4 | 2.4 | 1.2 |
| ICU admission | 57 | 5,454 | 104.5 |  | 9.0 | 9.0 | 32.4 |
| ICU with >=one SMM | 57 | 3,722 | 153.1 |  | 10.2 | 10.2 | 37.8 |
| ICU with no SMM | 0 | 1,732 | 0.0 |  | 6.4 | 6.4 | 20.8 |

SMM denotes severe maternal morbidity as previously defined by the Canadian Perinatal Surveillance System.^16^

Figure 3a. Temporal trends in maternal ICU admissions


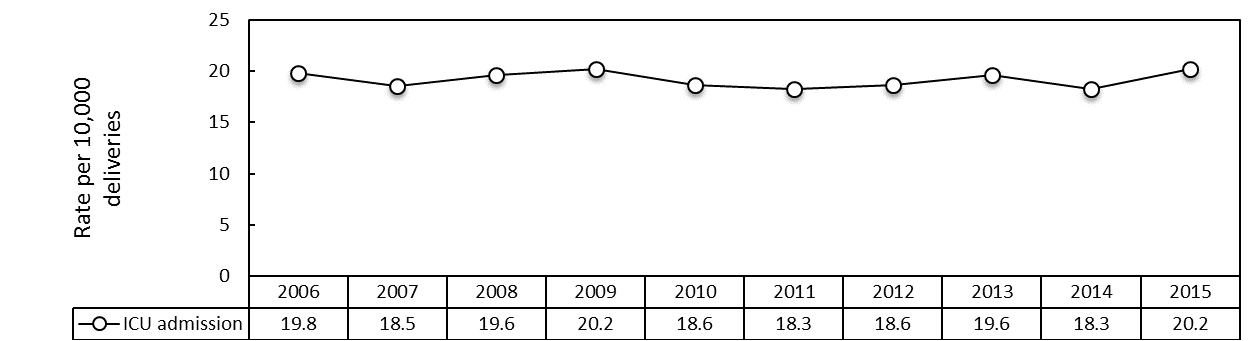


Figure 3b. Rates of maternal ICU admissions by province/territory


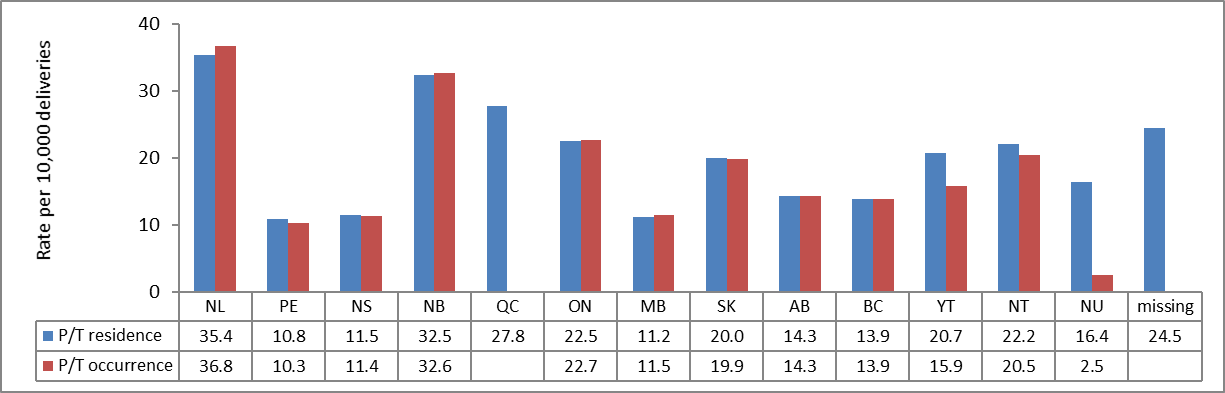


**Issue 4.** Should all women admitted to an intensive care unit (ICU) be considered cases of severe maternal morbidity or only those admitted to ICU for more than 24 hours?

**Results:** The frequency of maternal admission to ICU ranged from 18.3 to 20.2 per 10,000 deliveries. Case fatality rates and frequency of prolonged length of hospital stay were high in both women admitted to ICU for less than and more than 24 hours.

**Decision:** Retain all cases maternal admission to ICU as cases of severe maternal morbidity.

Table 4. Case fatality rates among women admitted to the ICU for <=24 and >24 hours, Canada (excluding Quebec), 2006-2015.

| Morbidity status | Case fatality rate (CFR) | | |  | Length of stay (LOS) | | |
| --- | --- | --- | --- | --- | --- | --- | --- |
|  | Deaths | Total deliveries | CFR/10,000 deliveries |  | Mean (all) | Mean (excl. deaths) | % >7 days (all) |
| No SMM | 12 | 2,801,128 | 0.04 |  | 2.3 | 2.3 | 1.1 |
| At least one SMM | 98 | 42,267 | 23.2 |  | 5.2 | 5.2 | 11.8 |
| All ICU | 57 | 5,454 | 104.5 |  | 9.0 | 9.0 | 32.4 |
| ICU <=24 hrs | 27 | 2,259 | 119.5 |  | 5.9 | 5.9 | 16.3 |
| ICU >24 hrs | 30 | 3,187 | 94.1 |  | 11.2 | 11.2 | 43.7 |

* 8 cases had missing information on ICU hours. SMM denotes severe maternal morbidity as previously defined by the Canadian Perinatal Surveillance System.^16^

Figure 4. Temporal trends in maternal admissions to the ICU
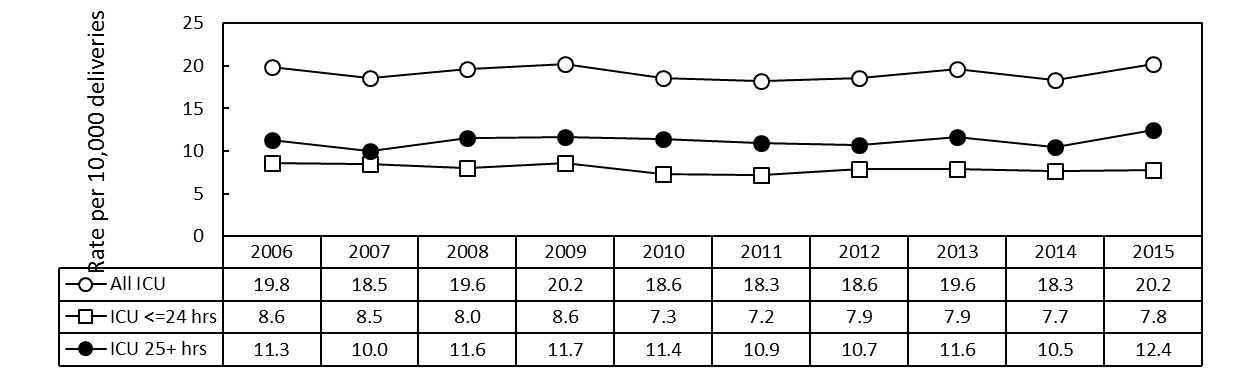


**Issue 5.** Should all women with uterine rupture be considered cases of severe maternal morbidity or only those with severe complications?

**Results:** The frequency of any uterine rupture was substantially greater than uterine rupture with PPH or uterine rupture with RBC transfusion, procedures to the uterus or hysterectomy. Case fatality rates were substantially higher for women with uterine rupture with RBC transfusion, procedures to the uterus or hysterectomy, while prolonged length of hospital stay was among all women with uterine rupture and PPH and those with uterine rupture with RBC transfusion, procedures to the uterus or hysterectomy.

**Decision:** Retain women with uterine rupture with RBC transfusion, procedures to the uterus or hysterectomy as cases of severe maternal morbidity.

Table 5. Cases of uterine rupture and associated case fatality rates and length of stay, Canada (excluding Quebec), 2006-2015.

| Morbidity status | Case Fatality Rate | | |  | Length of stay | | |
| --- | --- | --- | --- | --- | --- | --- | --- |
|  | Deaths | Total Cases | CFR/ 10,000 deliveries |  | Mean  (all cases) | Mean  (excl deaths) | % >7 days  (all cases) |
| No SMM | 12 | 2,801,128 | 0.04 |  | 2.34 | 2.34 | 1.1 |
| At least one SMM | 98 | 42,267 | 23.2 |  | 5.22 | 5.22 | 11.8 |
| Uterine rupture | 3 | 2,959 | 10.1 |  | 3.77 | - | 4.66 |
| Uterine rupture + PPH | 1 | 446 | 22.4 |  | 5.0 | - | 17.3 |
| Uterine rupture + (RBC transfusion or procedures to the uterus or hysterectomy) | 3 | 425 | 70.6 |  | 5.69 | - | 16.7 |

SMM denotes severe maternal morbidity as previously defined by the Canadian Perinatal Surveillance System.^16^

Figure 5. Temporal trends in uterine rupture and related morbidity.


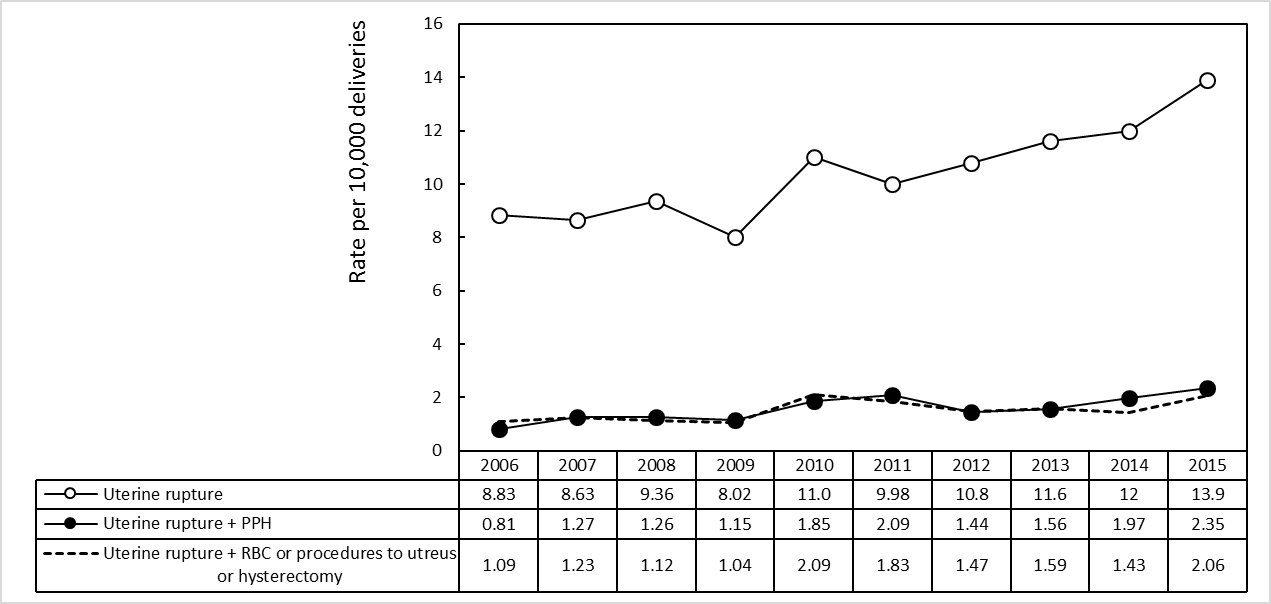


**Issue 6.** Should the previously used indicator, namely, postpartum hemorrhage with procedures for controlling bleeding including (a) embolization or ligation of pelvic vessels and (b) suturing of uterus occurring) be revised to include

1. Postpartum hemorrhage with a) RBC transfusion b) embolization or ligation of pelvic vessels, c) suturing of uterus or d) compression using uterine balloon (5.PC.91.HV) ?

**Result:** Adding red blood cell transfusion to the measure slightly increased the number of cases and captured a somewhat more

morbid population (C). There were no cases with compression using uterine balloon (5.PC.91.HV) before 2012, then an

increasing number of cases each year (D). However, the case fatality rate among such women was substantial and several cases

were identified with this code.

**Decision:** Retain E – Postpartum hemorrhage with (RBC transfusion or embolization or ligation of pelvic vessels or suturing of

uterus or compression using uterine balloon)

Table 6. Case fatality rates and length of hospitalization rates among women with postpartum hemorrhage and procedures to

control bleeding, Canada (excluding Quebec), 2006-2015.

| Morbidity status | Case fatality rate (CFR) | | |  | Length of stay (LOS) | | |
| --- | --- | --- | --- | --- | --- | --- | --- |
|  | Deaths | Total cases | CFR/10,000  deliveries |  | Mean (all) | Mean (excl. deaths) | % >7 days (all) |
| No SMM | 12 | 2,801,128 | 0.04 |  | 2.3 | 2.3 | 1.1 |
| At least one SMM | 98 | 42,267 | 23.2 |  | 5.2 | 5.2 | 11.8 |
| A | 5 | 3,201 | 15.6 |  | 5.7 | 5.7 | 12.9 |
| B | 5 | 1,506 | 33.2 |  | 7.9 | 7.9 | 23.3 |
| C | 6 | 3,451 | 17.4 |  | 5.9 | 5.9 | 14.1 |
| D | 8 | 1,987 | 40.3 |  | 7.2 | 7.2 | 19.8 |
| E | 11 | 4,845 | 22.7 |  | 5.5 | 5.5 | 12.0 |

A - embolization or ligation of pelvic vessels or suturing of uterus in combination with postpartum hemorrhage

B - embolization or ligation of pelvic vessels or suturing of uterus in combination with red blood cell transfusion

C - embolization or ligation of pelvic vessels or suturing of uterus in combination with postpartum hemorrhage or red blood

cell transfusion

D - embolization or ligation of pelvic vessels or suturing of uterus or compression using uterine balloon in combination with red blood

cell transfusion

E - embolization or ligation of pelvic vessels or suturing of uterus or compression using uterine balloon in combination with either

postpartum hemorrhage or red blood cell transfusion

SMM denotes severe maternal morbidity as previously defined by the Canadian Perinatal Surveillance System.^16^

Figure 6. Temporal trends in procedures to control bleeding


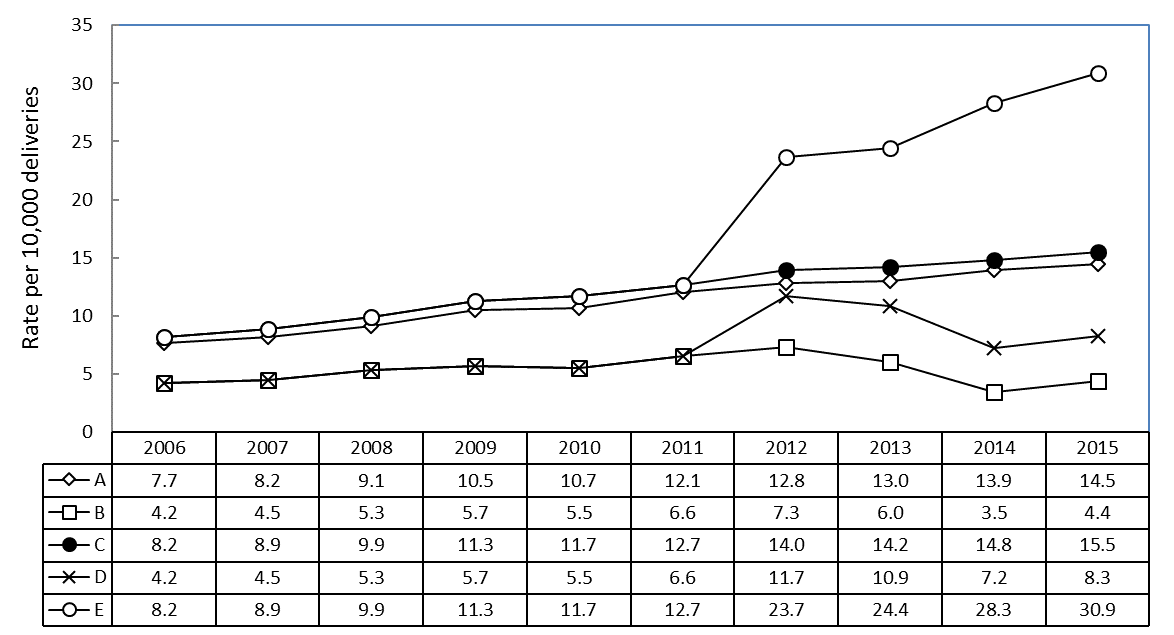


A - embolization or ligation of pelvic vessels or suturing of uterus in combination with postpartum hemorrhage

B - embolization or ligation of pelvic vessels or suturing of uterus in combination with red blood cell transfusion

C - embolization or ligation of pelvic vessels or suturing of uterus in combination with either postpartum hemorrhage or red blood cell

transfusion

D - embolization or ligation of pelvic vessels or suturing of uterus or compression using uterine balloon in combination with red blood cell

transfusion

E - embolization or ligation of pelvic vessels or suturing of uterus or compression using uterine balloon in combination with either postpartum

hemorrhage or red blood cell transfusion

**Issue 7.** Red blood cell (RBC) transfusions constituted the most frequent severe morbidity in the 2010 definition of severe maternal morbidity (647/100,000 compared with 1,380 per 100,000 deliveries for any severe maternal morbidity). Should RBC transfusions be considered an SMM per se or only in conjunction with other morbidity?

**Results:** RBC transfusions were often not associated with hemorrhage and other severe morbidity.

**Decision:** Use RBC transfusions as an indicator of severe maternal morbidity only in conjunction with other morbid conditions.

Figure 7. Temporal trends in transfusion of blood and blood components


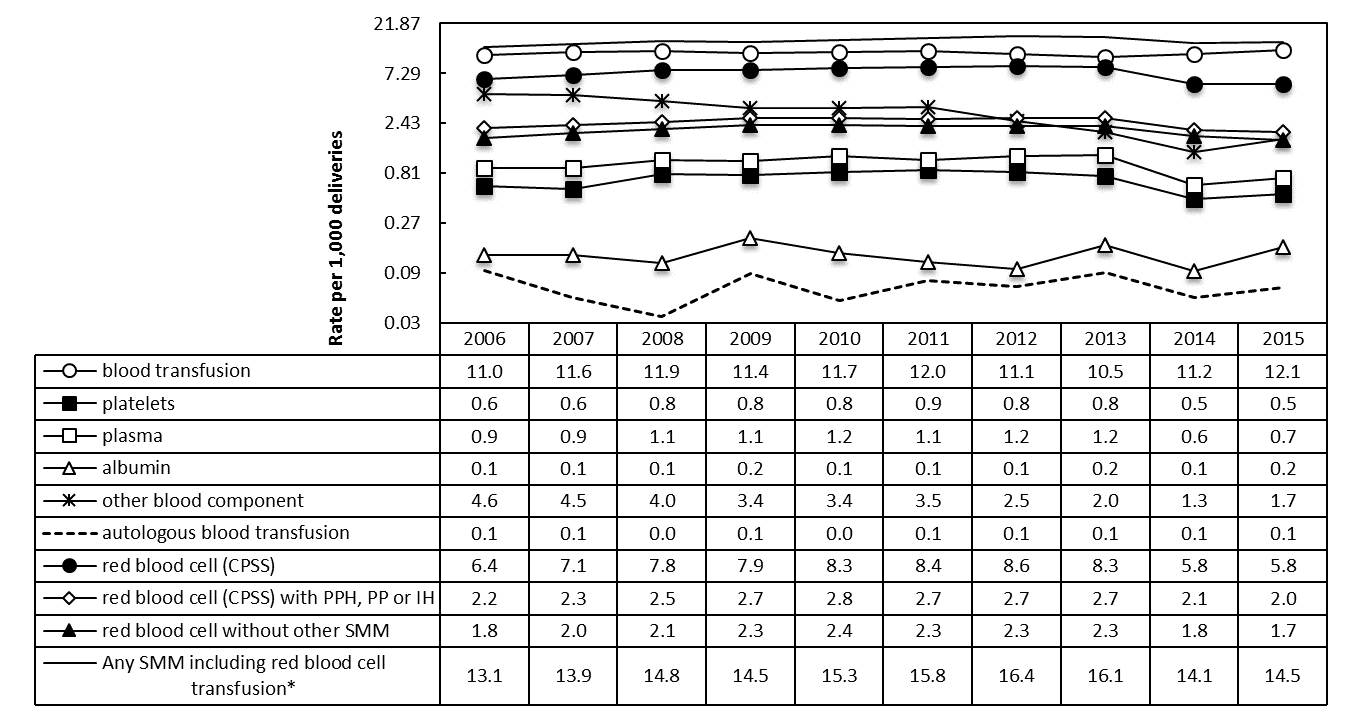


**Issue 8.** Do all hysterectomy procedures constitute a severe maternal morbidity?

**Results:** The frequency of hysterectomy for postpartum hemorrhage was approximately 0.5 per 1,000 deliveries postpartum and this was about half as frequent as subtotal hysterectomy. The latter was identified using the code 1.RM.89.LA-GX, while total hysterectomy open approach was identified using code 1. RM.89.LA (in the absence of 1.PL.74, 1.RS.74 and 1RS.80 which are codes for bladder neck suspension, concomitant suspension of vaginal vault and pelvic floor repair, respectively). Note that hysterectomy codes were also employed with uterine rupture.

**Decision:** Retain caesarean hysterectomy, total hysterectomy and subtotal hysterectomy as cases of severe maternal morbidity.

Table 8. Case fatality rates and length of hospitalization among women with hysterectyomy, Canada (excluding Quebec), 2006-2015.

| Morbidity status | Case fatality rate (CFR) | | |  | Length of stay (days) | | |
| --- | --- | --- | --- | --- | --- | --- | --- |
|  | Deaths | Total cases | CFR/10,000  deliveries |  | Mean  (all) | Mean (excl.  Deaths) | % >7 days (all) |
| No SMM | 12 | 2,801,128 | 0.04 |  | 2.3 | 2.3 | 1.1 |
| At least one SMM | 98 | 42,267 | 23.2 |  | 5.2 | 5.2 | 11.8 |
| Caesarean hysterectomy | <5 | 824 | <60.7 |  | 11.2 | - | 38.7 |
| Hysterectomy – Open approach | 10 | 567 | 17.6 |  | 10.5 | 10.7 | 43.7 |
| Subtotal hysterectomy | 10 | 2,672 | 3.7 |  | 5.1 | 5.1 | 14.3 |

SMM denotes severe maternal morbidity as previously defined by the Canadian Perinatal Surveillance System.^16^

Figure 8. Temporal trends in hysterectomy subtypes


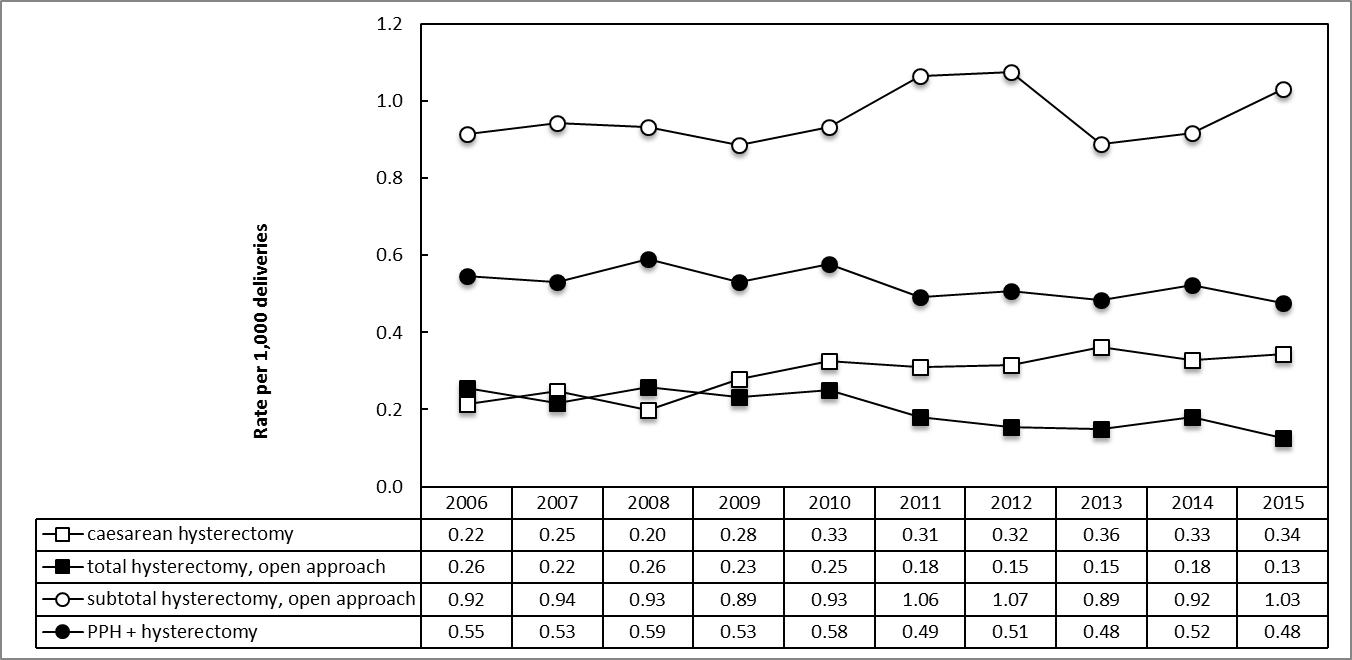


**Issue 9:** Should acute fatty liver (AFL) be considered a severe maternal morbidity?

**Results:** The frequency of AFL increased especially between 2008 and 2009 (likely a coding change). However, rates of AFL with plasma or RBC transfusion increased more modestly. Case fatality and prolonged length of hospital stay suggested that AFL was not a severe major maternal morbidity, although both AFL with plasma transfusion and AFL with plasma/RBC transfusion were severely morbid conditions.

**Decision:** Include AFL with plasma or RBC transfusion as a severe maternal morbidity.

Table 9. Case fatality rates and length of hospitalization rates among women acute fatty liver, Canada (excluding Quebec), 2006-2015.

| Morbidity status | Case fatality rate (CFR) | | |  | Length of stay (days) | | |
| --- | --- | --- | --- | --- | --- | --- | --- |
|  | Deaths | Total cases | CFR/10,000  deliveries |  | Mean  (all) | Mean (excl.  Deaths) | % >7 days (all) |
| No SMM | 12 | 2,801,128 | 0.04 |  | 2.3 | 2.3 | 1.1 |
| At least one SMM | 98 | 42,267 | 23.2 |  | 5.2 | 5.2 | 11.8 |
| AFL | 6 | 12,505 | 4.8 |  | 3.1 | 3.1 | 3.5 |
| AFL with plasma transfusion | 5 | 84 | 595.2 |  | 12.7 | 11.8 | 56.0 |
| AFL with plasma or RBC transfusion | 5 | 236 | 211.9 |  | 9.7 | 9.3 | 33.5 |

SMM denotes severe maternal morbidity as previously defined by the Canadian Perinatal Surveillance System.^16^

Figure 9. Temporal trends in acute fatty liver and acute fatty live with transfusion


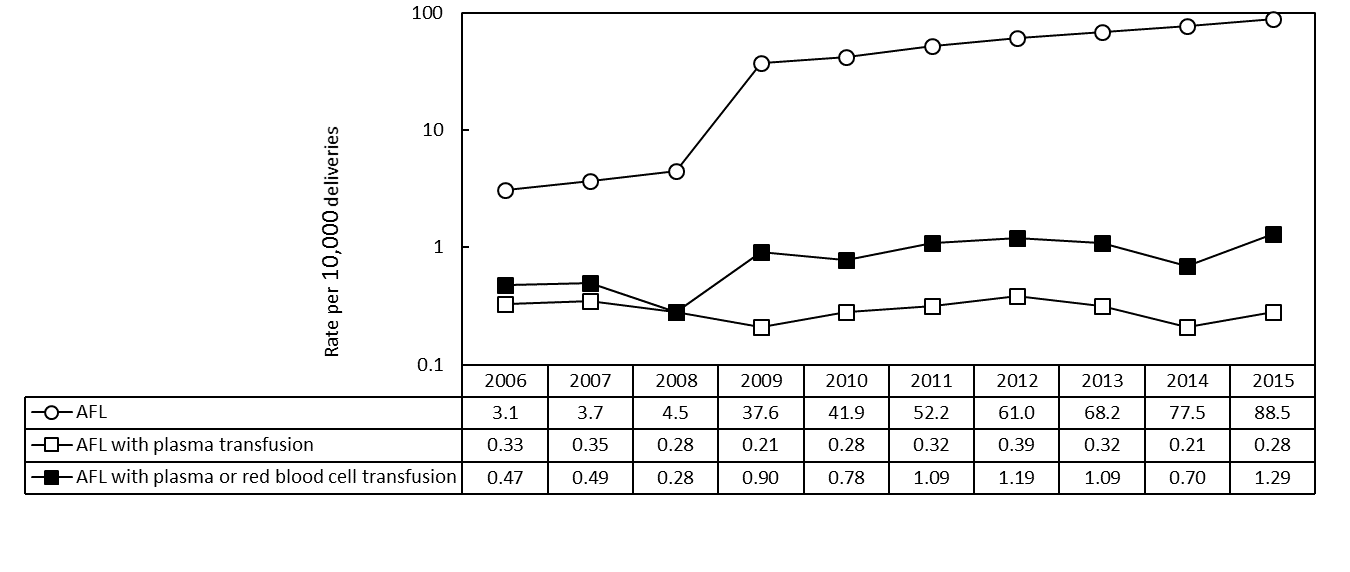


**Issue 10.** How will inclusion of severe pre-eclampsia and HELLP syndrome diagnoses affect severe maternal morbidity rates?

**Results:** The introduction of severe pre-eclapmsia and HELLP syndrome codes in 2012 altered the profile pattern of pre-eclampsia subtypes. The temporal trends in these subtypes are essentially incomparable before and after 2012. Note large fall in mild pre-eclampsia (moderate and severe pre-eclampsia cases were included in this category prior to 2012).

**Decision:** Include severe preeclampsia and HELLP syndromes as components of severe maternal morbidity (with the caveat that temporal trends in preeclampsia subtypes will not be comparable before and after 2012).

Table 10. Case fatality rates and length of hospitalization rates among women severe pre-eclampsia and HELLP syndrome, Canada (excluding Quebec), 2012-2015

Note: SMM and No SMM numbers and rates are for the period 2006-2015.

| Morbidity status | Case fatality rate (CFR) | | |  | Length of stay (days) | | |
| --- | --- | --- | --- | --- | --- | --- | --- |
|  | Deaths | Total cases | CFR/10,000  deliveries |  | Mean  (all) | Mean (excl.  Deaths) | % >7 days (all) |
| No SMM | 12 | 2,801,128 | 0.04 |  | 2.3 | 2.3 | 1.1 |
| At least one SMM | 98 | 42,267 | 23.2 |  | 5.2 | 5.2 | 11.8 |
| Severe pre-eclampsia | <5 | 2,927 | <17.1 |  | 6.3 | - | 33.0 |
| HELLP syndrome | <5 | 3,124 | <16.0 |  | 5.3 | - | 22.4 |

SMM denotes severe maternal morbidity as previously defined by the Canadian Perinatal Surveillance System.^16^

Figure 10. Temporal trends in pre-eclampsia and HELLP subtypes


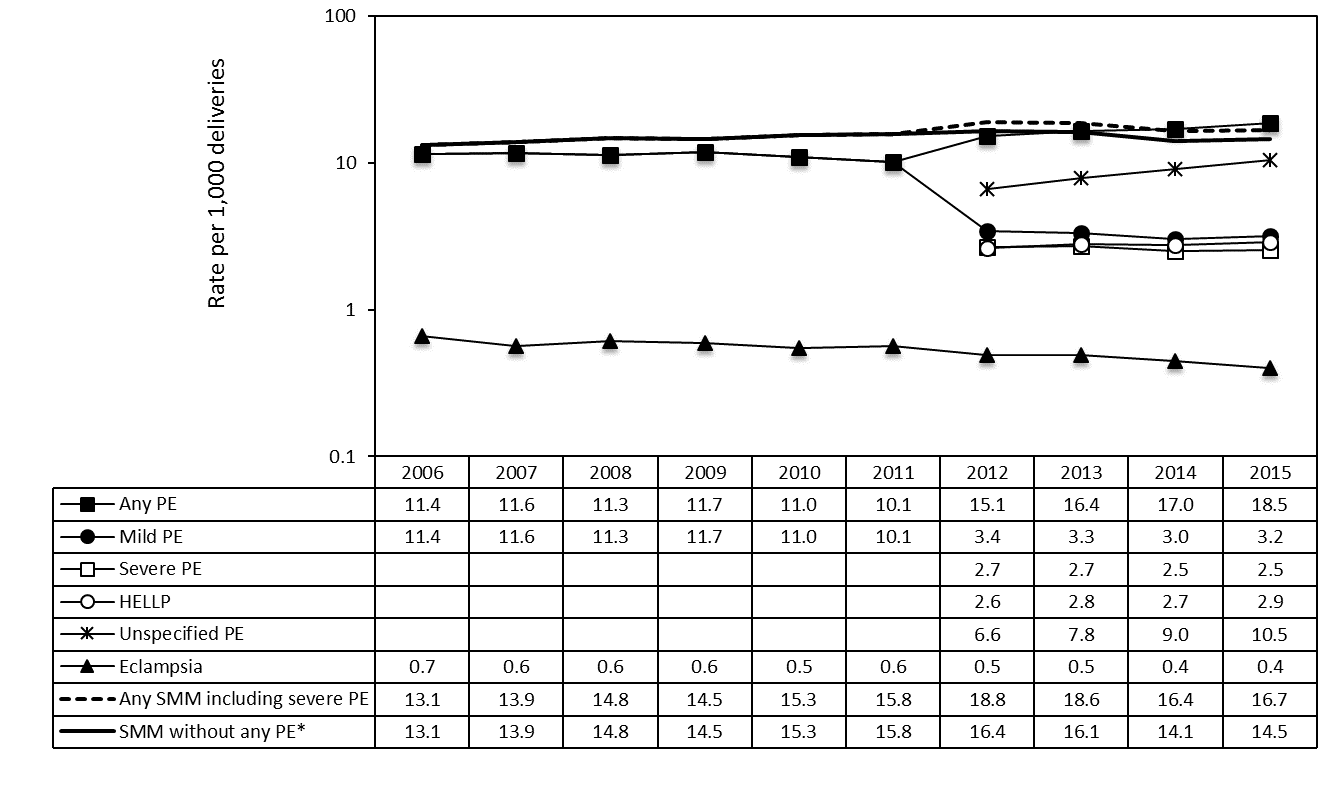


**Issue 11.** The 2010 composite severe maternal morbidity indicator identified cardiac arrest, cardiac failure, myocardial infarction and pulmonary edema using the following codes

O89.1 - Cardiac complications of anesthesia during the puerperium (includes cardiac arrest or failure due to anesthesia)

O74.2 - Cardiac complications of anesthesia during labour and delivery (includes cardiac arrest or failure due to anesthesia)

O75.4 - Other complications of obstetric surgery and procedures which includes

Cardiac arrest or failure following surgery/procedures

Cerebral anoxia following surgery/procedures

Conditions in N99.0 (post-procedural renal failure), N99.8 (other post-procedural disorders of the genitourinary

system) and N99.9 (post-procedural disorders of the genitourinary system unspecified).

I21-I22 - Acute myocardial infarction (I21) and subsequent myocardial infarction (I22)

I46 - Cardiac arrest

I50 - Heart failure

J81 - Pulmonary edema

This is problematic because the code O75.4 includes post-procedural renal failure and other and unspecified post-procedural disorders of the genitourinary system. Is it possible to specifically identify cardiac arrest, cardiac failure, myocardial infarction and pulmonary edema?

**Results:** Code O75.4 is the most frequently occurring diagnosis of those listed above. Cardiac arrest cases are better identified using a combination of ICD-10 CA and CCI (procedure codes).

**Decision:** Remove O75.4 from the list of severe maternal morbidity and instead use (in addition to the above)

I49.0 - Ventricular fibrillation and flutter

1.HZ.09 - Stimulation of the heart NEC which includes

Cardioversion (external), atrial

Conversion, cardiac [sinus] rhythm

Defibrillation, heart

Open cardiac massage

1.HZ.30 - Resuscitation, heart NEC

Cardio-cerebral resuscitation

Cardiopulmonary resuscitation

External cardiac massage

Figure 11a. Temporal trends in cardiac conditions


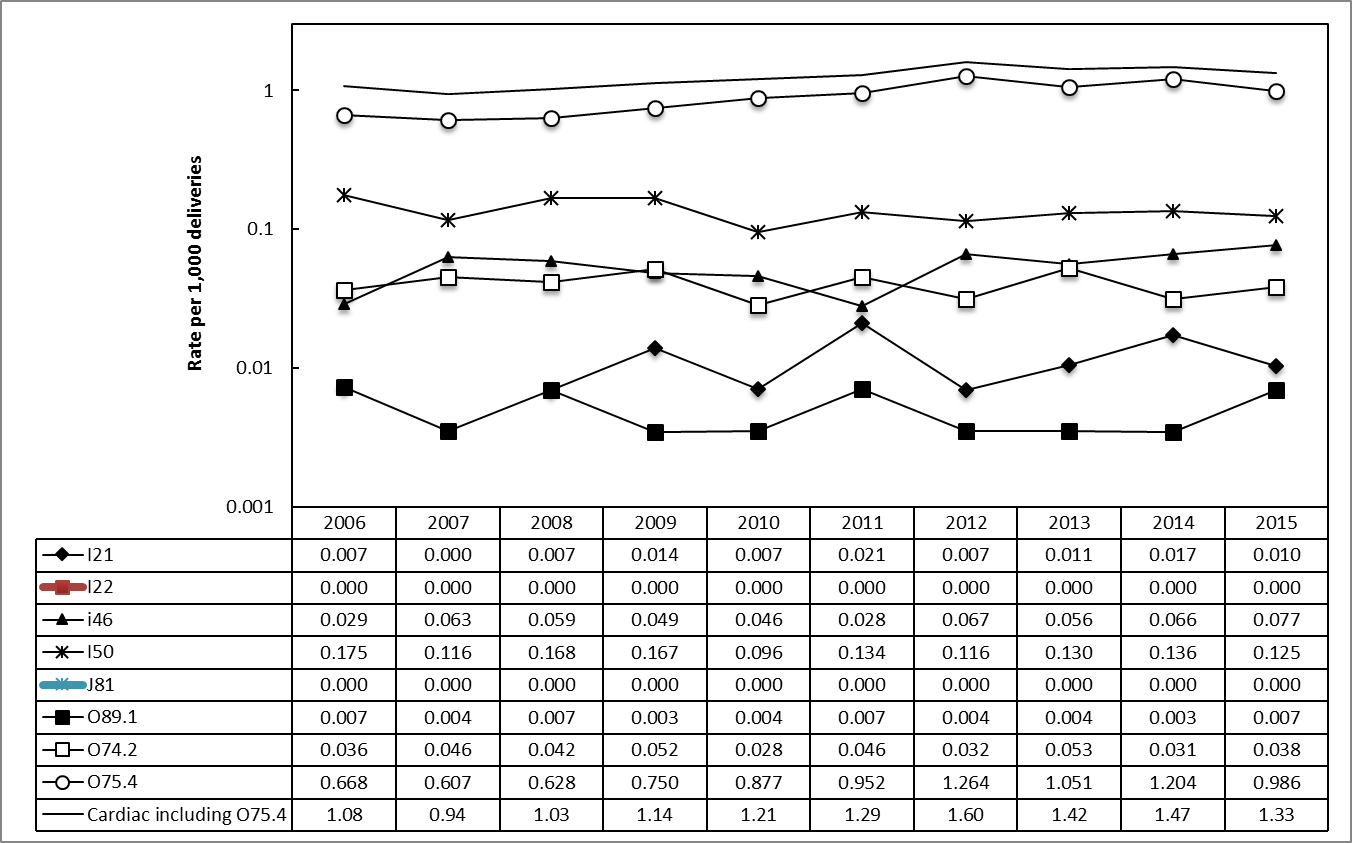


Table 11a. Case fatality rates and length of stay for women with cardiac morbidity, Canada (excluding Quebec), 2006-2015.

| Morbidity status | Case Fatality Rate | | |  | Length of stay | | |
| --- | --- | --- | --- | --- | --- | --- | --- |
|  | Deaths | Total Cases | CFR/ 10,000 deliveries |  | Mean  (all cases) | Mean  (excl deaths) | % >7 days  (all cases) |
| No SMM | 12 | 2,801,128 | 0.04 |  | 2.34 | 2.34 | 1.1 |
| At least one SMM | 98 | 42,267 | 23.2 |  | 5.22 | 5.22 | 11.8 |
| Cardiac codes Joseph et al. JOGC 2010 | 62 | 3,561 | 174.11 |  | 6.45 | 6.44 | 20.13 |
| Joseph et al without O75.4 | 47 | 1,210 | 388.43 |  | 9.16 | 9.22 | 38.35 |
| O75.4 | 15 | 2,351 | 63.8 |  | 5.01  6 | 5.01 | 10.75 |
| Joseph et al without 075.4 + Roberts ICD + Balki (ICD & procedures) | 67 | 1,775 | 377.46 |  | 8.03 | 8.11 | 32.73 |
| Joseph et al without 075.4 + Roberts ICD + Balki (ICD & procedures) + all other 1HZ09 | 68 | 1,793 | 379.25 |  | 8.02 | 8.10 | 32.57 |

Table 11b. Two algorithms for identifying cases of cardiac arrest.

| Joseph et al. JOGC 2010 | Diagnosis codes: O89.1, O74.2, I46 |
| --- | --- |
| Balki et al. *Anesth Analg* 2017 | Diagnosis codes : I460, I46.1, I46.9, I49.00, I49.01  Intervention codes : 1HZ09JAFS, 1HZ09LAFS, 1HZ09LACJ, 1HZ30 |

Table 11c. Frequency of cardiac arrest based on the 2 algorithms for identifying cases, Canada (excluding Quebec) 2006-2015.

Joseph et al (JOGC, 2010)

| **Proposed coding (Balki)** | No | Yes | Total |
| --- | --- | --- | --- |
| No | 2,843,011 | 108 (40.2%) | 2,843,119 |
| Yes | 81 | 161 (59.1%) | 242 |
| Total | 2,843,092 | 269 (100.0) | 2,843,361 |

Table 11d. Cases of cardiac arrest identified, cases fatality rates and length of stay, Canada (excluding Quebec) 2006-2015.

| Algorithm | No of cases | Case fatality  (No. and rate) | LOS>7 days | Mean LOS  (days) |
| --- | --- | --- | --- | --- |
| **Proposed coding (Balki)** | 242 | 65; 26.9% | 32.3% | 6.69 ± 6.52 |
| **SMM 2010 definition^16^** | 269 | 46; 17.1% | 22.3% | 5.57 ± 5.73 |

Figure 11b. Temporal trends in cases of cardiac arrest identified using 2 different algorithms


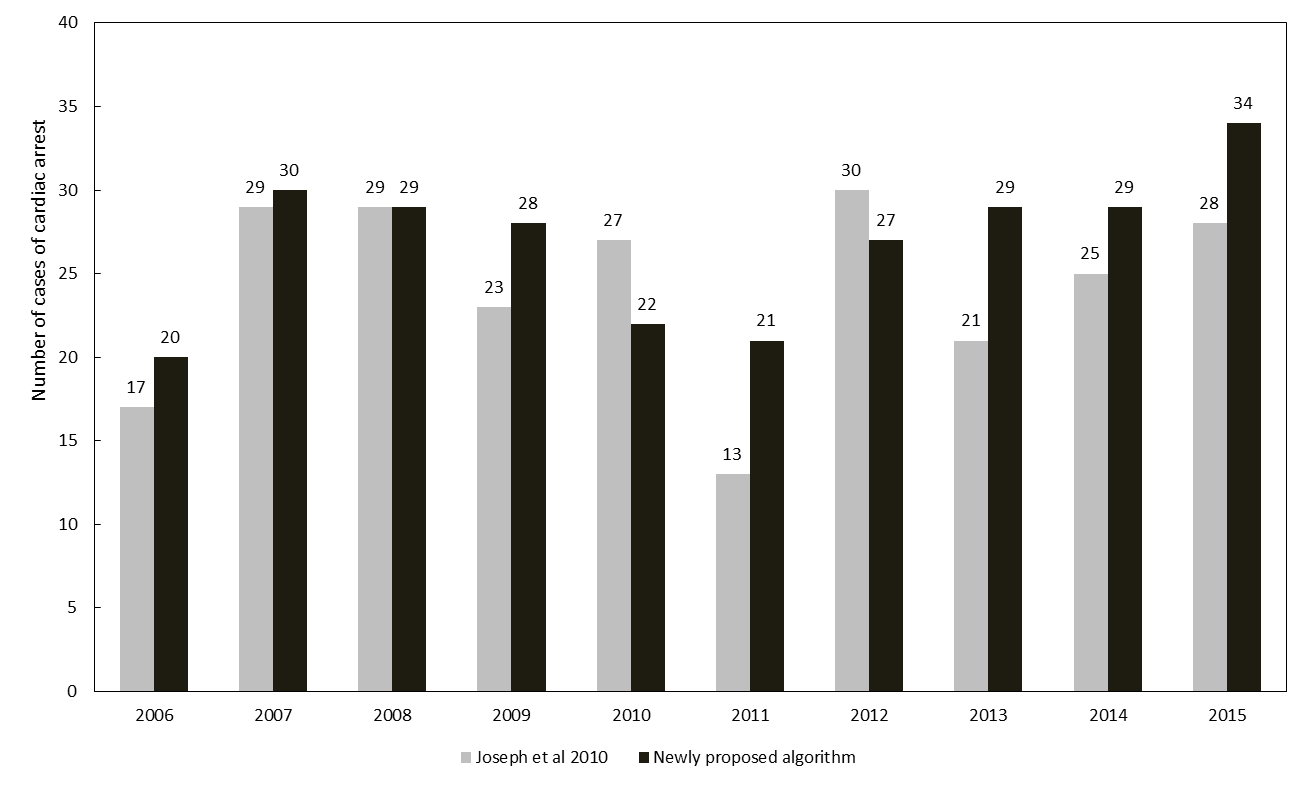


**Issue 12:** Should infection with human immunodeficiency virus (HIV) be considered a severe maternal morbidity (this was part of the 2010 severe maternal morbidity definition).

**Results:** The prevalence of HIV infection among women who delivered between 2006 and 2015 was 0.5 per 1,000 deliveries approximately. Universal availability of highly effective treatment for HIV infection in Canada means that this condition does not qualify as a severe maternal morbidity.

**Decision:** Remove asymptomatic HIV infection from the list constituting composite severe maternal morbidity and retain HIV disease as a severe maternal morbidity.

Table 12. Case fatality rates and length of hospitalization among women with HIV, Canada (excluding Quebec), 2006-2015.

| Morbidity status | Case fatality rate (CFR) | | |  | Length of stay (days) | | |
| --- | --- | --- | --- | --- | --- | --- | --- |
|  | Deaths | Total cases | CFR/10,000  deliveries |  | Mean  (all) | Mean (excl.  Deaths) | % >7 days (all) |
| No SMM | 12 | 2,801,128 | 0.04 |  | 2.3 | 2.3 | 1.1 |
| At least one SMM | 98 | 42,267 | 23.2 |  | 5.2 | 5.2 | 11.8 |
| HIV positive | 1 | 1,434 | 6.97 |  | 5.3 | - | 11.2 |
| Asymptomatic HIV infection | 0 | 1,320 | 0.0 |  | 5.2 | 5.2 | 11.0 |
| HIV disease | 1 | 114 | 87.7 |  | 5.8 | - | 13.2 |
| HIV positive, no SMM | 0 | 1,389 | 0.0 |  | 5.1 | 5.1 | 10.2 |

SMM denotes severe maternal morbidity as previously defined by the Canadian Perinatal Surveillance System.^16^

Figure 12. Temporal trends in HIV infection


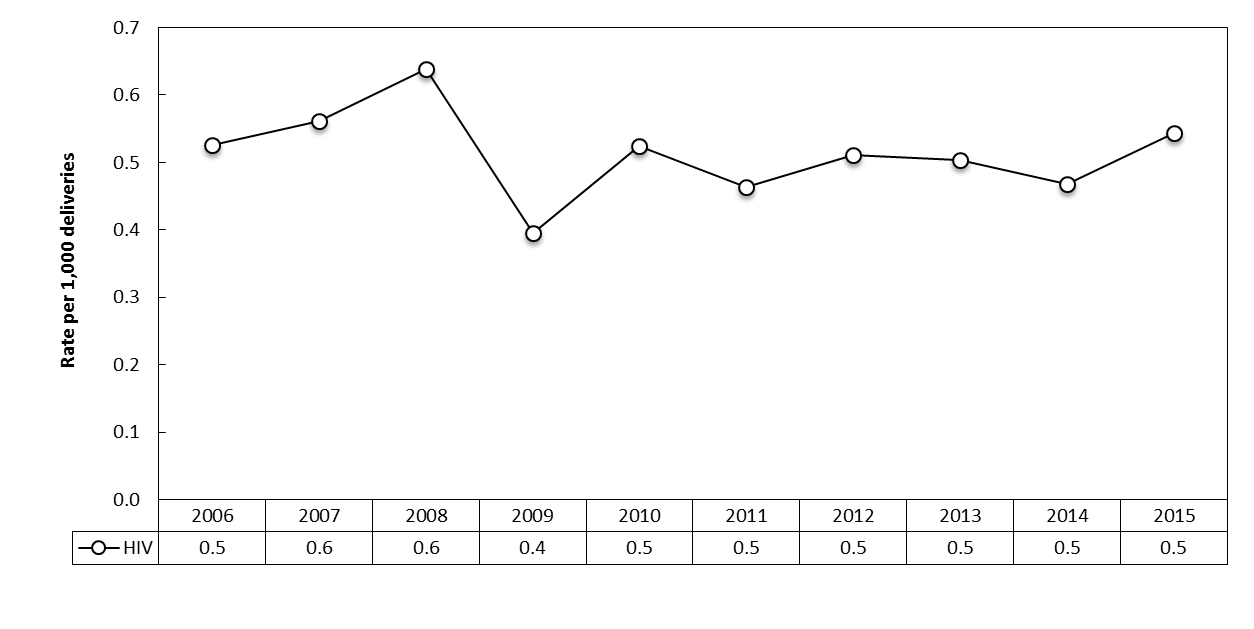


**Issue 13.** Should re-closure of caesarean wound or re-closure of caesarean wound in conjunction with red blood cell (RBC) transfusion be included in the list or severe maternal morbidity?

**Results:** Re-closure of caesarean wound was much more frequent than re-closure of caesarean wound with RBC transfusion.

**Decision:** Based on detail analysis of case fatality rates (not shown because of small numbers), re-closure of caesarean wound in conjunction with RBC transfusion is to be included in the list of composite severe maternal morbidity.

Table 13. Case fatality rates and length of hospitalization among women requiring re-closure of caesarean wound, Canada (excluding Quebec), 2006-2015.

| Morbidity status | Case fatality rate (CFR) | | |  | Length of stay (days) | | |
| --- | --- | --- | --- | --- | --- | --- | --- |
|  | Deaths | Total cases | CFR/10,000  deliveries |  | Mean  (all) | Mean (excl.  Deaths) | % >7 days (all) |
| No SMM | 12 | 2,801,128 | 0.04 |  | 2.3 | 2.3 | 1.1 |
| At least one SMM | 98 | 42,267 | 23.2 |  | 5.2 | 5.2 | 11.8 |
| Re-closure of CS wound | <5 | 5,636 | <8.87 |  | 3.95 | - | 4.2 |
| Re-closure of CS wound + RBC transfusion | 0 | 412 | 0.0 |  | 6.46 | 6.46 | 18.9 |

SMM denotes severe maternal morbidity as previously defined by the Canadian Perinatal Surveillance System.^16^

Figure 13. Temporal trends in cases requiring re-closure of caesarean wound


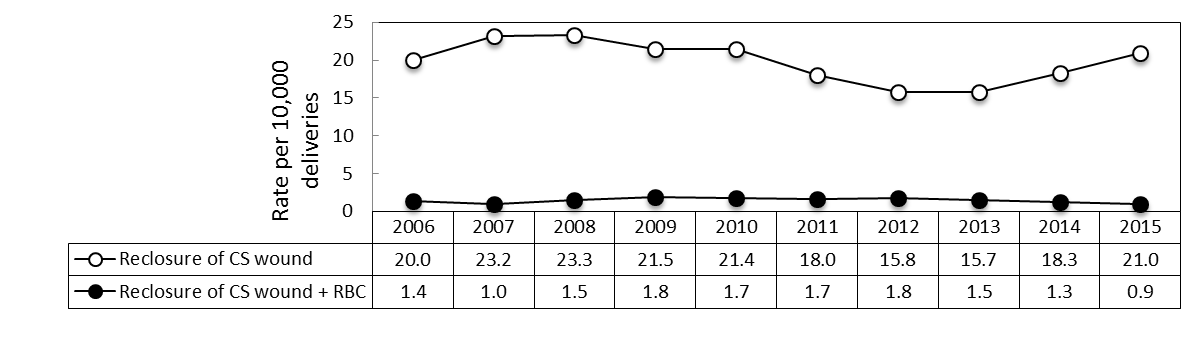


**Issue 14.** Should inversion of the uterus be considered a severe maternal morbidity?

**Results:** There were few cases of inversion of the uterus. Case fatality rates were high following uterine inversion in women having a vaginal delivery (not shown because of small numbers), although mean length of stay was not prolonged.

**Decision:** Include vaginal inversion of uterus in the list of severe maternal morbidity.

Table 14. Cases of uterine rupture and associated case fatality rates and length of stay, Canada (excluding Quebec), 2006-2015.

| Morbidity status | Case Fatality Rate | | |  | Length of stay | | |
| --- | --- | --- | --- | --- | --- | --- | --- |
|  | Deaths | Total Cases | CFR/ 10,000 deliveries |  | Mean  (all cases) | Mean  (excl deaths) | % >7 days  (all cases) |
| No SMM | 12 | 2,801,128 | 0.04 |  | 2.34 | 2.34 | 1.1 |
| At least one SMM | 98 | 42,267 | 23.2 |  | 5.22 | 5.22 | 11.8 |
| Vaginal inversion of uterus (corrected) | <5 | 289 | <173.0 |  | 2.92 | - | 0.35 |
| Inversion of uterus at caesarean (corrected) | 0 | 137 | 0.0 |  | 3.64 | - | 3.92 |

SMM denotes severe maternal morbidity as previously defined by the Canadian Perinatal Surveillance System.^16^

Figure 14. Temporal trends in inversion of uterus


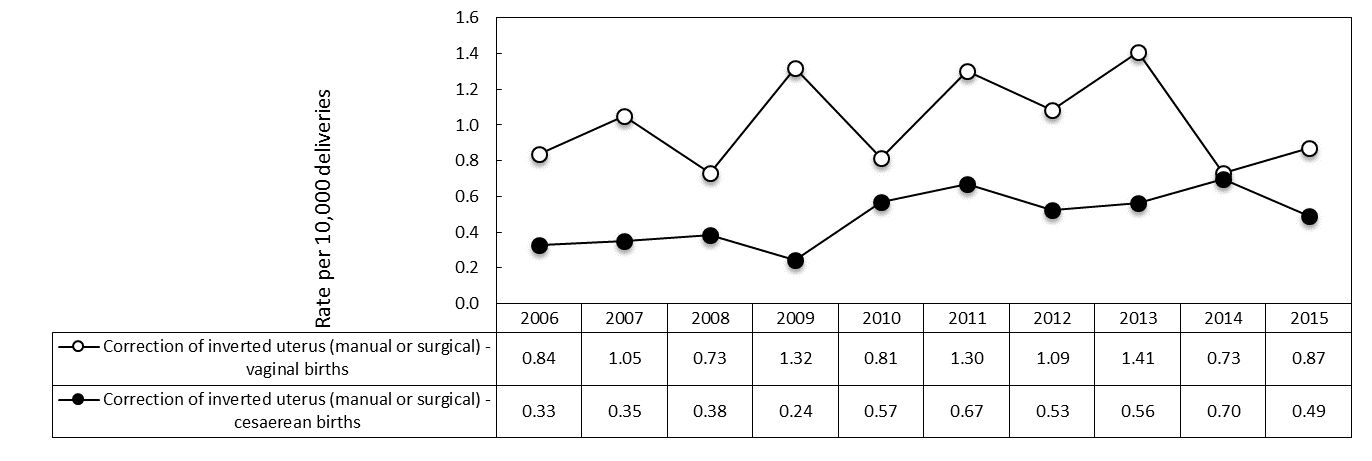


**Issue 15:** Should curettage of the uterus under anesthesia be included as a procedure indicating severe maternal morbidity?

**Result:** The frequency of curettage of the uterus was substantially higher than uterine curettage requiring RBC transfusion. Case fatality rates were higher in the latter group and length of stay was also longer. Anesthesia codes examined were not helpful. Most cases of curettage were carried out after delivery at term gestation.

**Decision:** Use curettage of the uterus in conjunction with RBC transfusion.

Table 16. Cases of uterine rupture and associated case fatality rates and length of stay, Canada (excluding Quebec), 2006-2015.

| Morbidity status | Case Fatality Rate | | |  | Length of stay | | |
| --- | --- | --- | --- | --- | --- | --- | --- |
|  | Deaths | Total Cases | CFR/ 10,000 deliveries |  | Mean  (all cases) | Mean  (excl deaths) | % >7 days  (all cases) |
| No SMM | 12 | 2,801,128 | 0.04 |  | 2.34 | 2.34 | 1.1 |
| At least one SMM | 98 | 42,267 | 23.2 |  | 5.22 | 5.22 | 11.8 |
| Curettage | <5 | 9,230 | <5.42 |  | 3.25 | - | 4.43 |
| Curettage + RBC transfusion | <5 | 2,165 | <23.1 |  | 4.29 | - | 6.84 |

SMM denotes severe maternal morbidity as previously defined by the Canadian Perinatal Surveillance System.^16^

Figure 15a. Temporal trends in uterine curettage


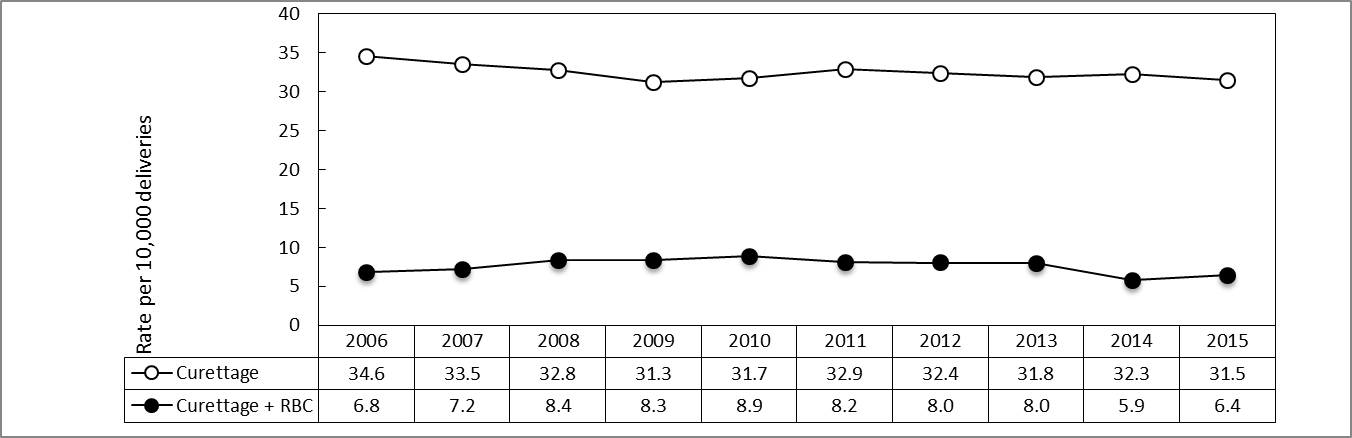


Figure 15b. Uterine curettage by gestational age at delivery (in weeks)


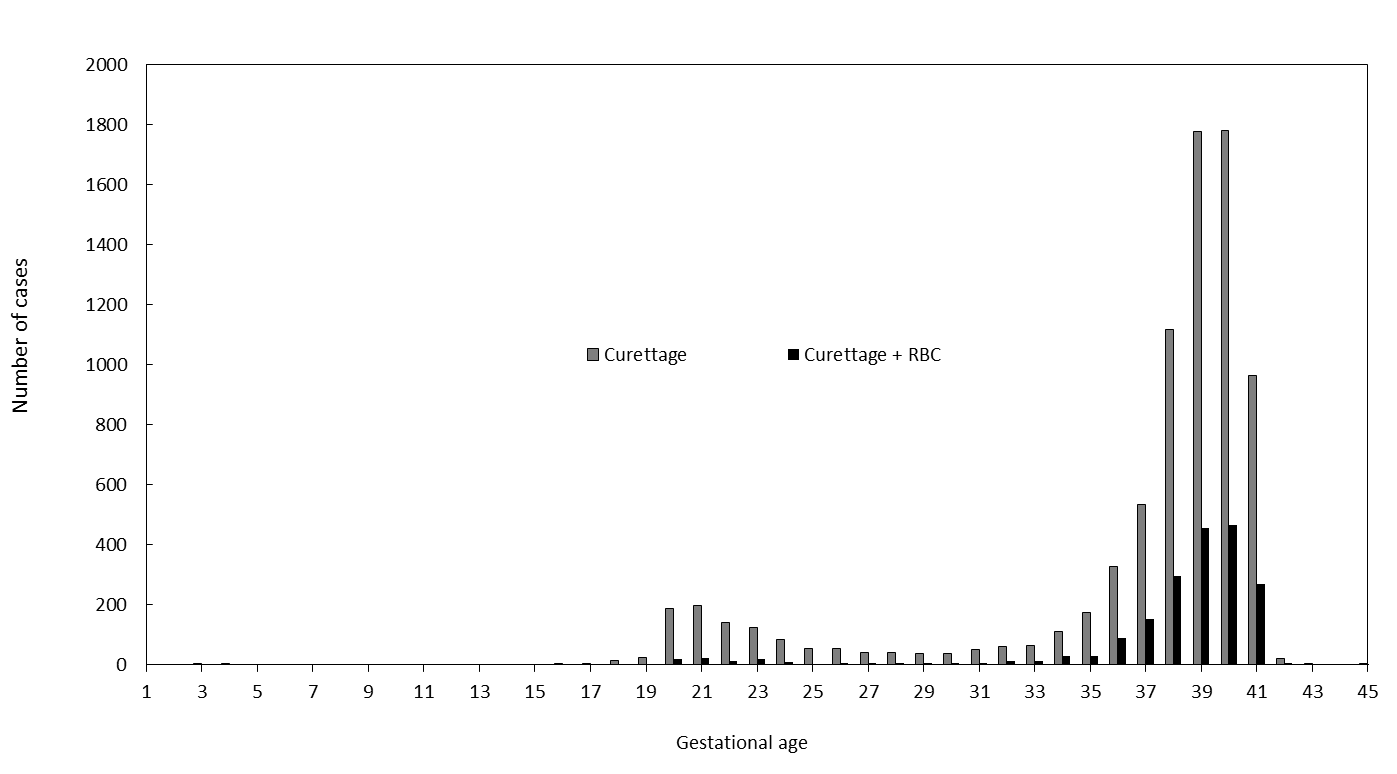


**Issue 16:** Should pre-existing hypertensive heart and pre-existing hypertensive renal disease be included as a component of severe maternal morbidity?

**Result:** The conditions were extremely rare in among women who delivered between 2006-2015 (2.3 per 100,000 deliveries). There were no associated death. The mean length of hospital stay was 4.5 days and 10.8% of such women had a prolonged length of stay (the latter was marginally lower than that among women with severe maternal morbidity per the 2010 defintion).

**Decision:** Pre-existing hypertensive heart and pre-existing hypertensive renal disease are to be excluded from the definition of severe maternal morbidity based on these estimates and on clinical grounds.

Table 16. Cases of pre-existing hypertensive and renal disease and associated case fatality rates and length of stay, Canada (excluding Quebec), 2006-2015.

| Morbidity status | Case Fatality Rate | | |  | Length of stay | | |
| --- | --- | --- | --- | --- | --- | --- | --- |
|  | Deaths | Total Cases | CFR/ 10,000 deliveries |  | Mean  (all cases) | Mean  (excl deaths) | % >7 days  (all cases) |
| No SMM | 12 | 2,801,128 | 0.04 |  | 2.34 | 2.34 | 1.1 |
| At least one SMM | 98 | 42,267 | 23.2 |  | 5.22 | 5.22 | 11.8 |
| Hypertensive and renal disease | 0 | 65 | 0.0 |  | 4.5 | 4.5 | 10.8 |

SMM denotes severe maternal morbidity as previously defined by the Canadian Perinatal Surveillance System.^16^

**Issue 17.** Should cardiomyopathy be included as a component of severe maternal morbidity?

**Result:** Cardiomyopathy was rare in among women who delivered between 2006-2015 (605 cases). The case fatality rates was substantial and the frequency of prolonged hospital stay was high as well.

**Decision:** Retain cardiomyopathy as a severe maternal morbidity.

Table 17. Case fatality rates and length of hospitalization among women with cardiomyopathy, Canada (excluding Quebec), 2006-2015.

| Morbidity status | Case fatality rate (CFR) | | |  | Length of stay (days) | | |
| --- | --- | --- | --- | --- | --- | --- | --- |
|  | Deaths | Total cases | CFR/10,000  deliveries |  | Mean  (all) | Mean (excl.  Deaths) | % >7 days (all) |
| No SMM | 12 | 2,801,128 | 0.04 |  | 2.3 | 2.3 | 1.1 |
| At least one SMM | 98 | 42,267 | 23.2 |  | 5.2 | 5.2 | 11.8 |
| Cardiomyopathy | <5 | 605 | <66.1 |  | 6.5 | - | 33.2 |

SMM denotes severe maternal morbidity as previously defined by the Canadian Perinatal Surveillance System.^16^

**Miscellaneous conditions**

Table 18. Case fatality rates (CFR) and length of stay (LOS) for selected morbidity codes, Canada

(excluding Quebec), 2006-2015.

|  | Case fatality rate | | |  | Length of stay | | |
| --- | --- | --- | --- | --- | --- | --- | --- |
|  | Deaths | Total cases | CFR / 10,000 deliveries |  | Mean  (all cases) | Mean  (excl deaths) | % >7 days  (all cases) |
| No SMM | 12 | 2,801,128 | 0.04 |  | 2.3 | 2.34 | 1.1 |
| At least one SMM | 98 | 42,267 | 23.2 |  | 5.2 | 5.22 | 11.8 |
| Incisional hematoma | |  |  |  |  |  |  |
| Hematoma | 0 | 665 | 0.0 |  | 5.7 | 5.65 | 17.6 |
| Hematoma with RBC | 0 | 133 | 0.0 |  | 7.5 | 7.50 | 33.1 |
| Acute abdomen | <5 | 239 | <209.2 |  | 11.4 | - | 36.0 |
| Anaesthesia complications | |  |  |  |  |  |  |
| SMM 2010 defintion^16^ | <5 | 360 | <138.9 |  | 4.7 | - | 9.2 |
| Roberts et al | <5 | 294 | <170.1 |  | 5.0 | - | 10.2 |
| Status asthmaticus | <5 | 56 | 892.9 |  | 6.4 | - | 14.3 |

SMM denotes severe maternal morbidity as previously defined by the Canadian Perinatal Surveillance System.^16^

Note on additional analyses

Other analyses were carried out including examination of the diagnoses and procedures associated with the women who died but did have a severe maternal morbidity (per the 2010 SMM definition^16^), procedures and diagnoses associated with women admitted to ICU who did not have a severe maternal morbidity (per the 2010 SMM defintion^16^), etc. These are not shown because of (small number) data suppression issues.
